# A Multiscale Simulation and Experimental Study on the Microfog Evolution and Dust Control Mechanism Driven by Ultrasonic Atomization

**DOI:** 10.1021/acsomega.5c11400

**Published:** 2026-01-30

**Authors:** Zhongan Jiang, Mingli Si, Fabin Zeng, Ming Wang, Yapeng Wang, Ya Chen, Guoliang Zhang, Wei Ma, Linquan Tong

**Affiliations:** a School of Resources and Safety Engineering, 12507 University of Science and Technology Beijing, Beijing 100083, China; b School of Safety Engineering, 71039 North China Institute of Science and Technology, Langfang 065201, China; c College of Environment and Safety Engineering, Qingdao University of Science and Technology, Qingdao 266042, China; d NHC Key Laboratory for Engineering Control of Dust Hazard, Beijing 102308, China

## Abstract

To address coal dust pollution arising during belt transportation in open-pit coal mine preparation plants, this study systematically elucidates the evolutionary dynamics of microdroplets and their dust suppression mechanisms under ultrasonic atomization. First, a theoretical analysis clarified the fundamental principles and dominant factors governing ultrasonic atomization. Subsequently, a combination of macroscopic spray experiments and microscopic numerical simulations was employed to investigate the coupled effects of gas pressure *P*_g_ and liquid pressure *P*_l_ on the gas–liquid flow ratio *Q*_l_/*Q*_g_ and droplet velocity *v*. Power-law correlations were established as *Q*_l_/*Q*_g_ = 6.16­(*P*_l_/*P*_g_)^−1.4^ and *v* = 0.176­(*P*_l_/*P*_g_)^−0.067^. Results indicate that the droplet concentration exhibits a radially symmetric unimodal distribution and decays monotonically along the axial direction. The dust suppression efficiency was found to be positively correlated to the airflow rate, particle size, and effective action distance. For near-field rapid capture of respirable dust, operating conditions with “high gas pressure–medium-to-low liquid pressure” (*P*_g_ ≥ 0.6 MPa, *P*_l_ = 0.1–0.3 MPa) were optimal, whereas for surface wetting and areal coverage scenarios, moderately elevated liquid pressure (*P*_l_ = 0.4–0.5 MPa) combined with medium gas pressure (*P*_g_ = 0.30–0.45 MPa) proved more favorable. Field applications demonstrated that under optimal conditions, the swirling ultrasonic atomizer achieved dust suppression efficiencies of approximately 80% for total dust and 75% for respirable dust. This research unveils the multiscale mechanisms of ultrasonic atomization for dust control and provides both theoretical insight and practical guidance for developing low-water, high-efficiency dust mitigation technologies in mining operations.

## Graphical abstract

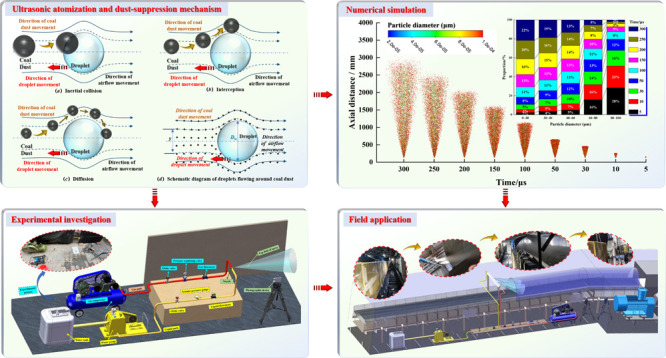

## Highlights

1) A power-law model links *P*_l_/*P*_g_ ratio with *Q*_l_/*Q*_g_ and droplet velocity.
2) Dust suppression efficiency is jointly regulated by gas and liquid flows.
3) The gas–liquid momentum ratio was identified as a key factor for atomization.
4) Optimal atomization achieves 80% total and 75% respirable dust control.

## 1 Introduction

The coal industry constitutes a fundamental pillar of national economic growth and social development; however, under conditions of high-intensity extraction and complex ventilation environments, coal dust exposure remains the predominant occupational health hazard and is closely linked to the latent risk of catastrophic accidents such as dust explosions.^1^ According to data released by the National Health Commission of the People’s Republic of China, a total of 12,087 new cases of occupational diseases were reported nationwide in 2023, of which 9051 were cases of pneumoconiosis, accounting for 74.88%. The majority of these patients were engaged in coal mining operations and had been subjected to long-term exposure to respirable dust.^2^ At present, micrometer-scale atomization technology represents the most effective and cost-efficient spray-based approach for controlling respirable dust. This efficacy arises from the dimensional similarity between micrometer-sized droplets and respirable particulates, which significantly enhances the probability of collision and capture. Nevertheless, owing to their inherently low inertia, micrometer-sized droplets are highly susceptible to air-current disturbances in confined underground mines or along conveyor belts in coal preparation plants, thereby impeding their capacity to achieve optimal dust suppression performance.^3–5^

Ultrasonic atomization nozzles have attracted considerable attention in both agricultural and industrial applications.^6^ At their core, the nozzle–resonance chamber system transforms pneumatic or mechanical energy into high-frequency acoustic perturbations, which, in concert with the shear-induced breakup of internally mixed gas flows, generate a micromist field characterized by “small droplet size, narrow spectrum, high number density, and spatial uniformity.” Such droplet fields markedly enhance the efficiency of inertial impaction, geometric interception, and liquid-bridge coalescence in the capture of respirable dust. Optimization of the nozzle design has primarily focused on two dimensions. First is the compact structure and loss reduction, achieved through shortening the mixing chamber, refining the throat–cavity volume matching, and adjusting flow-channel impedance to minimize internal energy dissipation while improving integration.^7^ Second is the systematic design of geometrical and swirling parameters, wherein variations in the swirl angle, nozzle divergence angle, and orifice size are investigated to regulate droplet spectra and velocity fields.^8^ Experimental and numerical evidence demonstrates that the Sauter mean diameter (SMD) decreases with increasing swirl angle, yielding a more concentrated radial distribution;^9^ likewise, enlarging the divergence angle contributes to a reduction in mean droplet size.^10^ These insights establish the technical foundation for achieving scale and momentum matching between droplets and dust particles in coal dust mitigation.

To meet the demands of coal dust control, droplet groups with small diameters and narrow spectra within the range of *St* = *O*(1) are more likely to achieve an optimal balance between high capture efficiency and low energy consumption.^11,12^ Consequently, extensive numerical simulations and atomization experiments have been conducted to advance spray-based dust suppression technologies. Saeedipour et al.^13^ employed a coupled Euler–Lagrange approach to simulate the primary breakup of liquid jets, obtaining distributions of droplet velocities and sizes. Charinpanitkul and Tanthapanichakoon^14^ established a predictive model for inertial interception efficiency, revealing that nozzles producing smaller droplets with narrower size distributions delivered superior dust suppression performance. Zhalehrajabi et al.^10^ analyzed the influence of nozzle geometry on mean droplet size and demonstrated that increasing the divergence angle effectively reduced the average droplet diameter. Simakov^15^ numerically simulated the two-phase flow generated by atomizing nozzles using unsteady compressible flow differential equations coupled with droplet mass-transfer equations, thereby obtaining axial and radial distributions of droplet size, air velocity, and impurity concentration in the spray field. Han et al.^16^ experimentally investigated the effect of water supply pressure on the atomization characteristics and dust suppression efficiency of internal-mixing air-atomizing nozzles, finding that both droplet size and velocity increased with rising water pressure. Xie et al.,^17^ using a 3D FiberPDA system, examined the spatial distribution of atomization performance, observing that mean droplet size initially decreased and then increased with increasing axial distance from the nozzle exit; meanwhile, the proportion of 15–70 μm droplets first increased and subsequently declined, while mean droplet velocity and flux consistently decreased, indicating significant nonuniformity in axial atomization parameters. Furthermore, Tian et al.^18^ proposed a method utilizing a 3D PIV high-speed imaging system combined with a particle size analysis platform to characterize droplet groups with specific properties. Their study revealed that as the momentum and kinetic energy ratios between droplets and dust particles increased, the coupling effect exhibited nonlinear variation. Notably, dust particles with diameters in the 20–50 μm range played a decisive role in the suppression efficiency of internal-mixing pneumatic atomizing nozzles. Collectively, these investigations systematically elucidate the coupling mechanisms between atomization characteristics and dust suppression efficiency, thereby providing robust engineering and technical foundations for effective dust control in coal mines.

In response to dust control demands, researchers have conducted extensive engineering studies on airflow fields, dust fields, and droplet fields, while also developing a variety of spray-based suppression systems suitable for mining operations such as fully mechanized longwall and tunneling faces.^5,19^ Beck et al.,^20^ under simulated longwall airflow conditions, performed comprehensive experiments on hollow-cone, full-cone, air-atomizing, and hydraulic nozzles. Their findings revealed that higher spray pressures produced droplets of smaller size and greater velocity, thereby achieving superior suppression efficiency, even under airflow disturbance. Wang et al.^21^ proposed an integrated foam–fine mist dust suppression technology, which, when applied to tunneling faces, exhibited highly effective dust removal owing to the multistage refinement of the water mist. Zhou et al.,^22^ based on the coupling mechanisms of atomized flow fields and turbulent air curtains, developed an airflow–droplet collaborative dust control method tailored for mechanized excavation faces. Nie et al.^23^ advanced a novel wind–fog combined suppression technology, which was deployed in large-height longwall faces, and identified the optimal operating parameters of an integrated negative-pressure dust removal system, thereby significantly enhancing overall suppression efficiency. Wang et al.,^24^ through a combination of numerical simulation and field trials, demonstrated that reducing the surface tension of the spray solution substantially improved atomization performance; when surface tension was reduced to 61.9 mN/m, droplet size decreased noticeably, and field applications reported total dust suppression efficiencies of up to 90%, with respirable dust reduction rates reaching 85%. Yang et al.,^25^ integrating numerical modeling and experimental validation, determined the optimal parameter scheme for tunnel wind-fog coupled dust control technology. Their results highlighted airflow velocity as the dominant factor, and a regression model with prediction errors within 5% was established, offering a reliable predictive capability for practical applications.

Existing research on ultrasonic atomization has largely concentrated on nozzle structures and atomization performance characterization while offering an insufficient systematic explanation of the mechanisms by which droplets capture dust and the dominant factors governing this process. Moreover, the spatial distribution of spray two-phase flow fields and their coupling relationship with capture efficiency remain inadequately understood. To address these gaps, this study adopts the dust suppression mechanism of droplets as its central theme. At the macroscopic scale, controlled experiments were conducted under adjustable gas and liquid supply conditions to quantify the effects of liquid and gas pressures on gas flow characteristics, liquid flow characteristics, atomization angle, and the effective range of ultrasonic atomization. At the microscopic scale, numerical simulations were employed to elucidate the formation mechanisms of key field variables, including droplet velocity, droplet size, and droplet concentration, and to construct an optimal parameter domain map for atomization operating conditions. Finally, targeting the application scenario of the belt corridor in the Zhundong open-pit coal mine preparation plant in Xinjiang, an integrated ultrasonic atomization dust suppression system was engineered and validated through field trials. The results demonstrated that the technology possesses a dual advantage of high efficiency and low water consumption, thereby underscoring its potential for large-scale implementation in mining operations.

The principal contributions of this study to research on ultrasonic dry fog and air-assisted spraying are as follows: (1) It elucidates the interaction mechanisms between gas–liquid momentum coupling and secondary atomization under varying gas–liquid supply conditions and systematically clarifies the governing influence of gas and liquid pressures on micrometer-scale mist generation and transport. (2) It establishes a multiscale modeling framework linking droplet size migration and velocity relaxation to dust suppression performance, thereby unifying microscopic droplet evolution with macroscopic suppression efficacy. (3) Through an integrated program of theory, experimentation, numerical simulation, and field verification, it proposes an optimal *P*_g_ and *P*_l_ parameter domain that enables highly efficient dust suppression under low water consumption, providing a robust foundation for engineering design and operational optimization.

## 2 Ultrasonic Atomization and Dust Suppression Mechanism

### 2.1 Ultrasonic Atomization Device and Principle Analysis

Ultrasonic atomization technology has been widely applied in industrial spraying, dust suppression, and related fields. Its fundamental principle lies in transforming liquid into ultrafine microdroplets through ultrasonic nozzles, thereby enhancing the atomization efficiency and improving droplet uniformity. In this study, an ADG SV882 (60°) ultrasonic dry-fog nozzle was employed as the core component of a gas–liquid coaxial internal-mixing, mechanically resonant ultrasonic atomization device, as shown in Figure 1. The apparatus primarily consists of a nozzle base, a guide tube, a gas–liquid mixing chamber, and a resonance chamber/atomizing head. The overall dimensions are approximately 75 × 12.5 mm (length × diameter) with a stainless steel 304/316 housing. Standard interfaces include a 1/4” gas inlet (KTS 10-02) and a 1/8” liquid inlet (SER 8-01).

**Figure 1:**
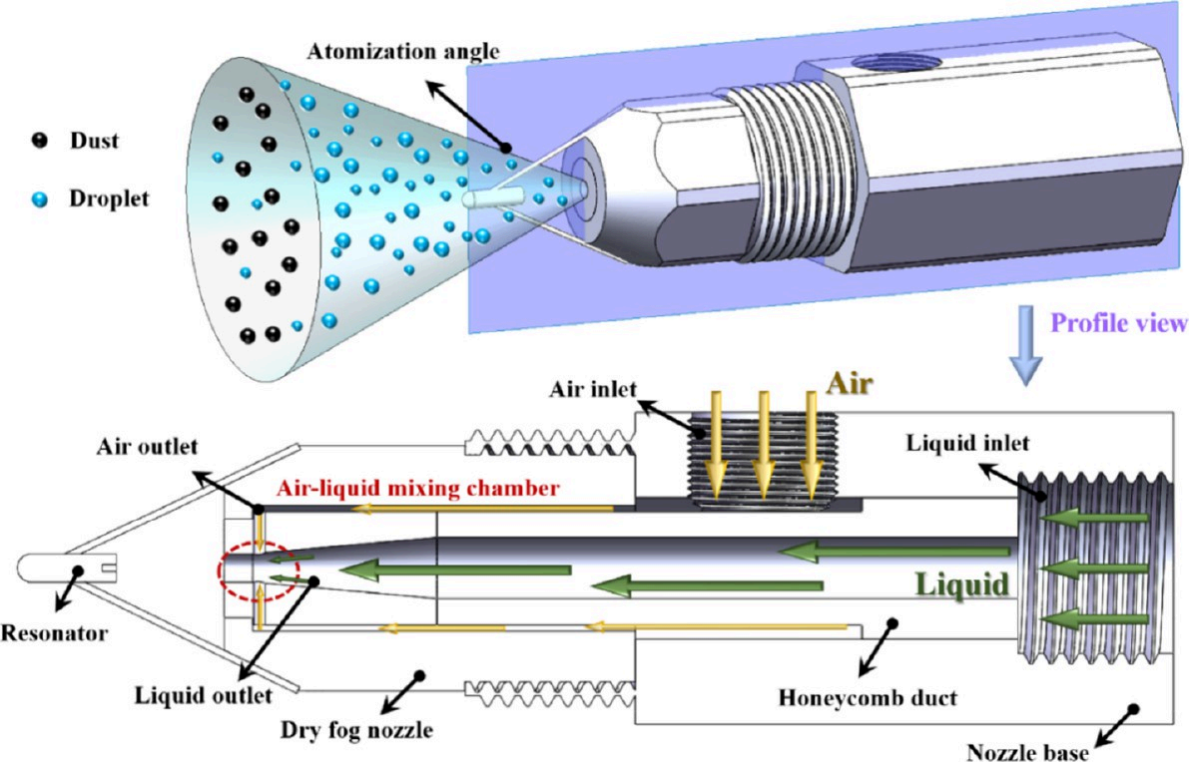
Schematic of the ultrasonic atomization device.

The nozzle operates on a coupled swirl–ultrasonic atomization mechanism. Compressed air enters the nozzle through a helical passage, generating a strong swirling flow field that imposes a primary shear-induced breakup on the liquid stream. The liquid is then subjected to oscillatory perturbations within the resonance chamber, undergoing secondary disintegration to produce a dry fog characterized by a narrow size distribution and high uniformity. Compared with conventional twin-fluid nozzles, the ADG SV882 achieves stable release of micrometer-scale droplets under low water consumption, thereby markedly enhancing inertial impaction, Brownian diffusion, and coalescence between droplets and particulates. This makes it extremely efficient in dust-capture applications while also providing an excellent water-saving performance.

### 2.2 Mechanistic Analysis of Ultrasonic Atomization for Dust Suppression

The capacity of a single droplet to capture dust particles is one of the core parameters determining the efficiency of spray-based dust suppression. As illustrated in Figure 2a–c, coal dust is continuously intercepted and removed through the combined actions of inertial impaction, interception, and diffusion. When a dust-laden airflow with relative velocity *U*_r_ passes around a small droplet, the surrounding air streamlines bend and flow around the droplet’s surface. Dust particles, however, owing to their inertia, cannot fully follow the streamlines; instead, they deviate from the airflow trajectory and shift toward the droplet. Particles approaching the droplet’s central axis eventually collide with its windward surface and are captured, as depicted in Figure 2d.

**Figure 2:**
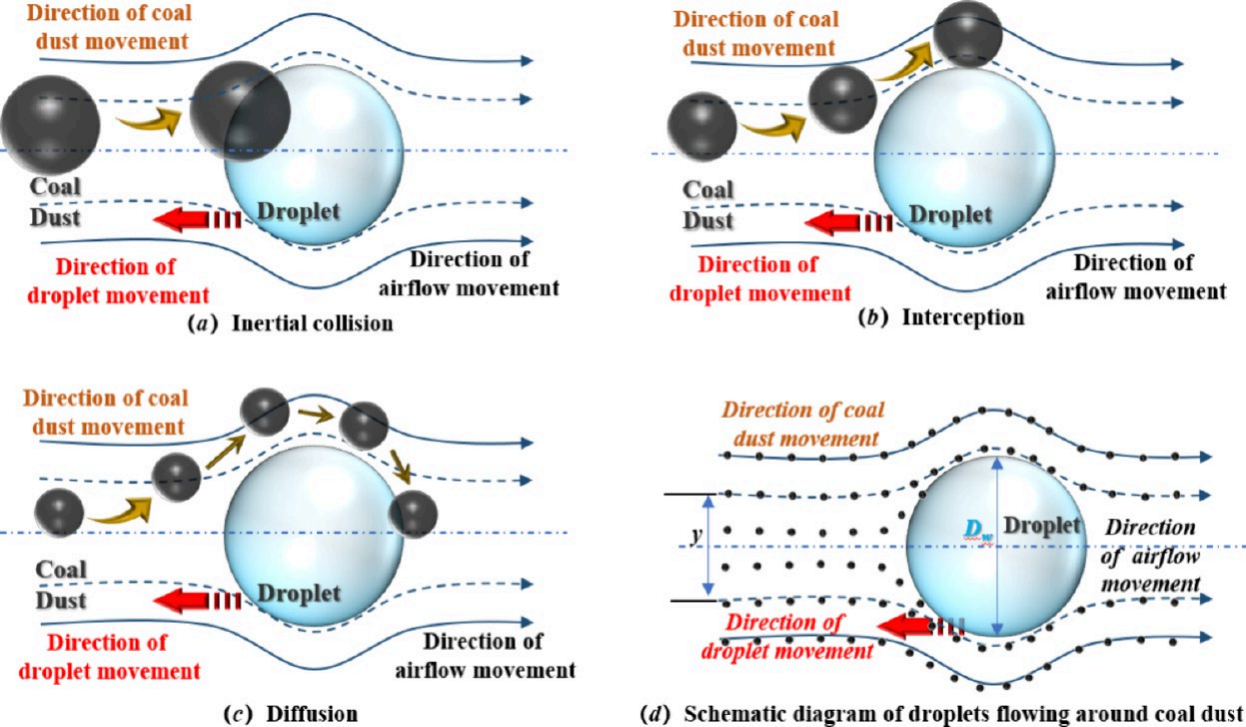
(a–d) Schematic of the inertial interaction between a single droplet and dust particles.

To construct an analytically tractable model of droplet-based dust suppression efficiency and to ensure that the theoretical derivations possess a clearly defined range of applicability, the following rational assumptions are adopted:^26^ (1) The local two-phase turbulent structures within the effective interaction zone are simplified to a quasi-steady gas-borne flow field, thereby allowing the relative motion between droplets and dust particles to be described using a continuum approximation; (2) dust particles are idealized as spherical, so that their dynamical response can be uniformly characterized by the Stokes number, consistent with the canonical form of inertial impaction theory; (3) given that the characteristic interaction time scale between droplets and dust is on the order of milliseconds and that the spray region maintains a high humidity, the influence of evaporation on particle size variation may be neglected; (4) to obtain a closed-form analytical expression for the collection efficiency, a characteristic mean relative velocity is employed in place of the instantaneous turbulent velocity, thereby enabling a statistically averaged treatment of the complex gas–liquid two-phase flow field.

During the relative motion between airflow and droplets, it is assumed that dust particles located within a streamline tube with a diameter of *y* can be entirely captured by the droplet. Accordingly, the capture efficiency *E* of an individual droplet can be expressed as the ratio of the streamline tube cross-sectional area to the projected area of the droplet, namely,

$$E=\frac{y^{2}}{D_{w}^{2}}$$ 1

Furthermore, assuming that dust particles are uniformly distributed in the air, with a number concentration *n* per unit volume, the number of dust particles *N* captured by a single droplet per unit time can be determined by combining the particle flux with the effective capture cross section of the droplet. This relationship is expressed as

$$N=nU_{r}\pi \frac{y^{2}}{4}=EnU_{r}\pi \frac{D_{w}^{2}}{4}$$ 2

By normalization, the ratio of the dust-capture rate of a single droplet to the volumetric airflow *Q*_g_ can be obtained, thereby establishing the droplet dust-capture kinetic model:

$$-\frac{dn}{dt}=EnU_{r}\pi \frac{D_{w}^{2}}{4Q_{g}}$$ 3

For a multidroplet system under nozzle atomization conditions, if the spray water flow rate is *Q*_l_, the total number of droplets can be derived from the relationship between the droplet volume and spray water volume to obtain $\frac{Q_{l}}{\pi D_{w}^{3}/6}$ . Extending the single droplet capture model to droplet groups can obtain the total dust-capture rate for the entire spray field, that is

$$-\frac{dn}{dt}=EnU_{r}\pi \frac{D_{w}^{2}}{4Q_{g}}\frac{Q_{l}}{\pi D_{w}^{3}/6}=\frac{3}{2}EnU_{r}\frac{Q_{l}}{Q_{g}D_{w}}$$ 4

If the cross-sectional area of the dust removal region is denoted as *A*_0_ and a segment of length d*x* is considered, with the dust-laden airflow and droplets moving in opposite directions at a relative velocity *U*_r_, then, within a time interval d*t*, the airflow traverses a distance d*x* and the dust mass concentration decreases by d*n*. Thus, the relation can be expressed as

$$U_{r}=\frac{dx}{dt}$$ 5

$$-\frac{dn}{dt}=-\frac{dn}{dx}\frac{dx}{dt}=-\frac{dn}{dx}U_{r}$$ 6

In conjunction with eq 4, the following expression is obtained:

$$dn=-\frac{3}{2}En\frac{Q_{l}}{Q_{g}D_{w}}dx$$ 7

Assuming that the effective action distance of the droplets is *L*, with the initial dust mass concentration in the region denoted as *n*_1_ and the postsuppression concentration as *n*_2_, then integrating both sides of eq 7 yields

$$\int_{n_{1}}^{n_{2}}\frac{dn}{n}=-\frac{3}{2}En\frac{Q_{l}}{Q_{g}D_{w}}\int_{0}^{L}dx$$ 8

The solution is thus obtained as

$$\frac{n_{2}}{n_{1}}=\exp\left(-\frac{3EQ_{l}L}{2Q_{g}D_{w}}\right)$$ 9

The dust suppression efficiency η is defined as the reduction in dust mass concentration divided by the inlet concentration, namely,

$$\eta =\frac{n_{1}-n_{2}}{n_{1}}=1-\frac{n_{2}}{n_{1}}=1-\exp\left(-\frac{3EQ_{l}L}{2Q_{g}D_{w}}\right)$$ 10

Considering only inertial effects, the dust-capture efficiency of a single droplet through inertial impaction is given by^4^

$$E={\left(\frac{K}{K+0.7}\right)}^{2}$$ 11

where *K* represents the dimensionless inertial parameter of particle motion, known as the Stokes number, defined as

$$Κ=\frac{U_{r}\rho_{p}D_{P}^{2}}{9\mu_{g}D_{w}}$$ 12

In this expression, ρ_P_ denotes the dust particle density (kg/m^3^), *D*_P_ represents the particle diameter (m), and μ_g_ is the dynamic viscosity of the gas (Pa s).

In the model, the relative velocity between the droplets and dust particles is approximated by the characteristic droplet velocity within the effective interaction region. Considering the pronounced velocity relaxation of dry fog in the near field, where droplet velocity rapidly decays to about 30–60% of the nozzle exit velocity downstream, and following the results of Jiang et al.^27^ and Ma and Kou,^28^ the characteristic relative velocity is taken as one-half of the nozzle exit velocity, expressed as

$$U_{r}=\frac{Q_{l}+Q_{g}}{2A_{0}}$$ 13

By combining eqs 9–13, we can obtain

$$\eta =1-\exp[-\frac{3Q_{l}L}{2Q_{g}D_{w}}\times {\left(\frac{(Q_{l}+Q_{g})\rho_{p}D_{P}^{2}}{(Q_{l}+Q_{g})\rho_{p}D_{P}^{2}+12.6\mu_{g}D_{w}A_{0}}\right)}^{2}]$$ 14

From eq 14, it follows that the dust suppression efficiency of droplets is primarily governed by the nozzle structure, the physical properties of the gas–liquid phases, the gas flow rate *Q*_g_, the liquid flow rate *Q*_l_, the gas–liquid flow ratio *Q*_g_/*Q*_l_, the particle size diameter *D*_P_, and the effective action distance *L* of the droplets.

#### 2.2.1 Influence of Gas–Liquid Flow Rates on Dust Suppression Efficiency

When the dust particle diameter (*D*_P_) is 10 μm and the effective action distance of droplets (*L*) is 4 m, Matlab was employed to plot the variation curves of droplet dust suppression efficiency with respect to changes in the water flow rate and airflow rate, as illustrated in Figure 3.

**Figure 3:**
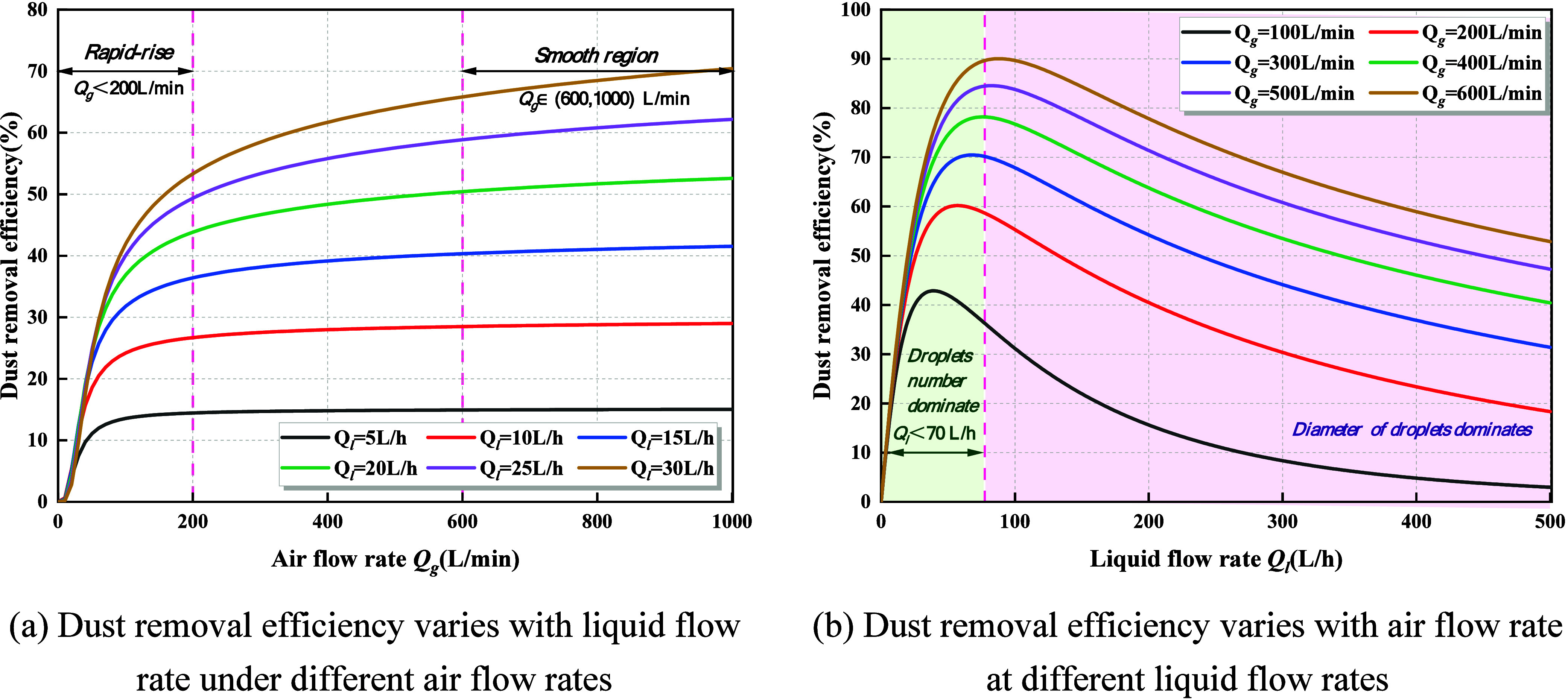
Effect of gas–liquid flow rates on dust removal efficiency. (a) Dust removal efficiency varies with the liquid flow rate under different airflow rates. (b) Dust removal efficiency varies with the airflow rate at different liquid flow rates.

(1) Under a constant water flow rate, the dust suppression efficiency exhibits a typical monotonic trend of a “rapid initial increase followed by saturation” with increasing airflow rate. As shown in Figure 3a, in the low airflow region (*Q*_g_ < 200 L/min), the momentum input from the gas phase is insufficient, resulting in weak droplet transport and dispersion and limited spatial coverage, which leads to low suppression efficiency. As the airflow rate increases, the enhanced turbulence intensity and higher gas–liquid relative velocity substantially improve droplet entrainment, conveyance, and spatial dispersion, thereby enlarging the overlap region between droplets and dust particles and causing a marked rise in suppression efficiency. When the airflow rate further increases to the medium–high range (*Q*_g_ ∈ (600, 1000) L/min), droplets can already penetrate the dust-laden region effectively and the capture process approaches saturation. Thus, additional increases in the airflow rate no longer produce significant improvements in droplet dispersion or transport, and the suppression efficiency gradually levels off. This indicates that gas-phase momentum is the primary driver governing droplet entrainment and effective coverage, but its enhancing effect diminishes once the critical turbulence intensity is reached.
(2) At a constant airflow rate, the dust suppression efficiency exhibits a typical “increase–then–decrease” nonmonotonic trend with respect to the water flow rate, corresponding to an optimal liquid supply range. As shown in Figure 3b, in the low water flow region (*Q*_l_ < 70 L/h), the droplet number flux increases significantly with the rising liquid flow rate, thereby enhancing the probability of droplet–dust collisions. Meanwhile, the atomization film becomes more stable and the spray coverage improves, leading to a rapid increase in suppression efficiency. However, when the water flow rate exceeds a critical threshold (*Q*_l_ > 100 L/h), the spray system enters a liquid-overloading regime. The excessive liquid supply weakens the breakup capacity of the airflow, resulting in enlarged droplet sizes, stronger inertial settling, and reduced flowability such that many large droplets precipitate prematurely in the near-field region. At the same time, the decline in the gas–liquid momentum ratio suppresses secondary breakup and weakens the fine mist zone, thereby reducing the concentration of effective suspended droplets within the dust-laden region. Consequently, the overall dust suppression efficiency decreases.

#### 2.2.2 Influence of Dust Particle Size on Dust Suppression Efficiency

Figure 4 illustrates the characteristic variation of dust suppression efficiency with particle size under a spray effective action distance of 4 m using an ultrasonic atomization nozzle. Across all operating conditions, dust removal efficiency increases sharply with particle size and gradually stabilizes once *D*_P_ falls within the range of 40–60 μm. This trend indicates that the spray exhibits a markedly higher capture efficiency for coarse particles, while its capacity to control respirable dust and PM_2.5_-level particulates remains limited.

**Figure 4:**
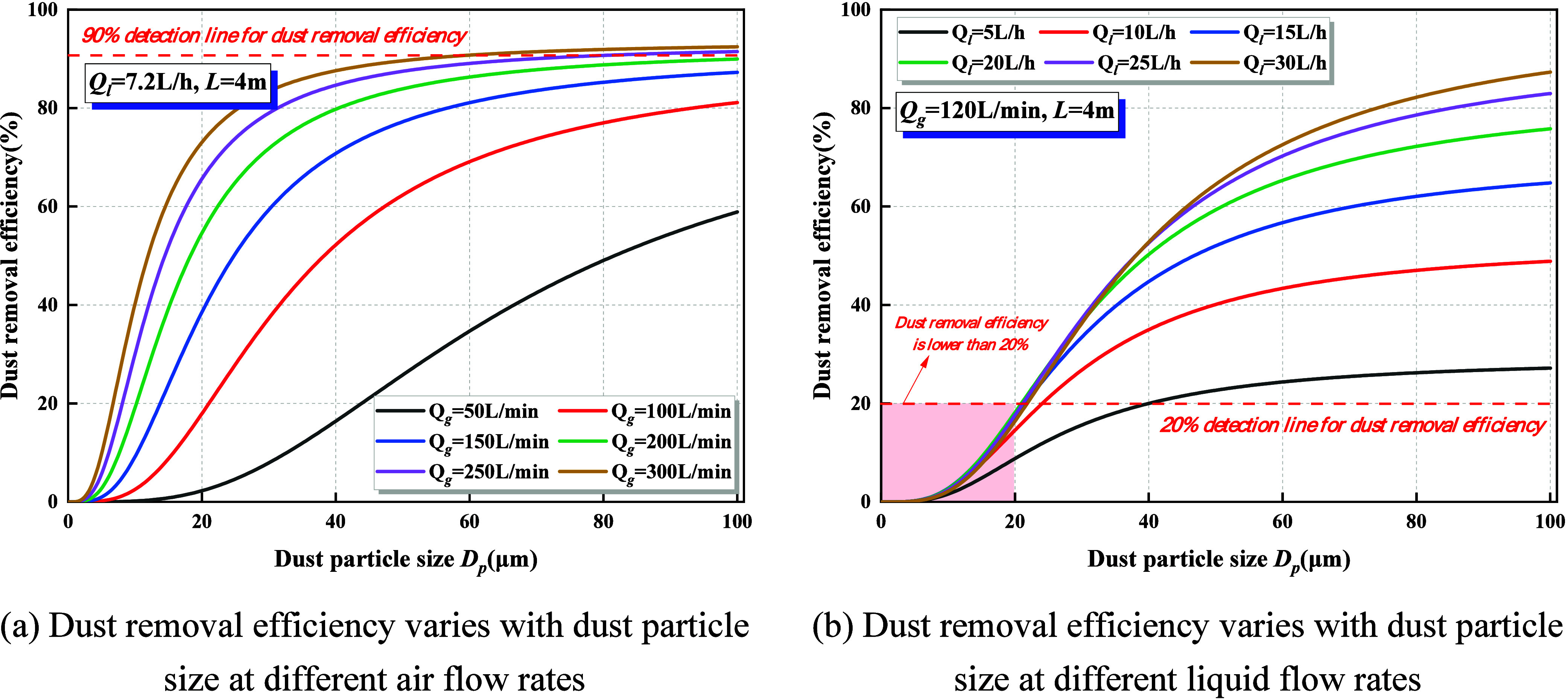
Effect of dust particle size on dust removal efficiency. (a) Dust removal efficiency varies with dust particle size at different airflow rates. (b) Dust removal efficiency varies with dust particle size at different liquid flow rates.

(1) At a constant water flow rate of *Q*_l_ = 7.2 L/h, increasing the airflow rate markedly enhances dust suppression efficiency, while the critical particle size at which efficiency saturates shifts toward smaller ranges. When *Q*_g_ = 300 L/min, the capture efficiency for particles larger than 40 μm approaches 90%, whereas under low airflow conditions, efficiency within the same size range remains considerably lower. This demonstrates that greater gas momentum strengthens droplet dispersion and transport, thereby promoting relative motion and spatial overlap between droplets and dust particles, which in turn substantially improve the capture efficiency.
(2) At a constant airflow rate of *Q*_g_ = 120 L/min, the liquid flow rate remains within the range of *Q*_l_ = 5–30 L/h, where the process is predominantly governed by the number of droplets. Within this interval, increasing the water flow generates a larger droplet population, thereby consistently enhancing the suppression efficiency. However, the collection efficiency for particles smaller than 20 μm remains below 20%. This is primarily because fine particles possess extremely low inertia, making it difficult for them to deviate from their original streamlines and collide effectively with droplets. In addition, their weak aerodynamic coupling with the droplets allows them to follow the airflow around the droplets rather than enter the droplet-impaction trajectory. Furthermore, for PM_2.5_ particles, Brownian motion-induced random migration tends to displace them away from the droplet-dominated inertial capture zone, further reducing their probability of effective collection.

#### 2.2.3 Influence of the Effective Distance on Dust Suppression Efficiency

Figure 5a,b presents the relationships between dust suppression efficiency and the effective action distance of spray droplets for an ultrasonic atomizing swirl nozzle at various gas–liquid flow conditions with a dust particle size of 10 μm. The analysis indicates that suppression efficiency (η) increases monotonically with the effective spray distance (*L*) and gradually approaches saturation. The overall curve exhibits a concave-downward profile, characterized by a steep initial slope and diminishing marginal gains in the far field.

**Figure 5:**
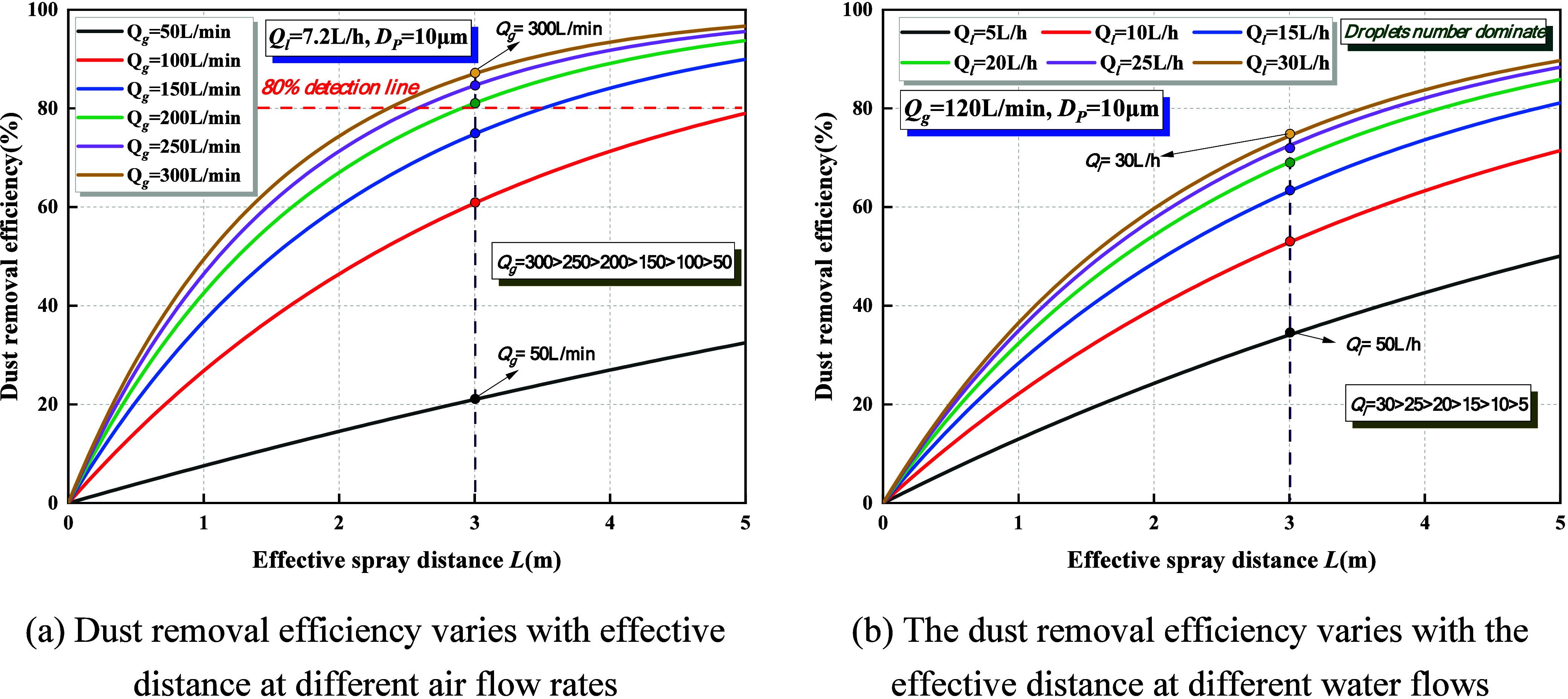
Effect of the effective distance on dust removal efficiency. (a) Dust removal efficiency varies with the effective distance at different airflow rates. (b) Dust removal efficiency varies with the effective distance at different water flows.

(1) At a fixed water flow rate, the efficiency curves shift upward as *Q*_g_ increases, while the characteristic distances (*L*, such as *L*_50_ for 50% efficiency or *L*_90_ for 90% efficiency) are markedly shortened. The ordering *Q*_g_ = 300 > 250 > 200 > 150 > 100 > 50 L/min holds consistently across all distances. Physically, a higher *Q*_g_ enhances gas-phase momentum and turbulence intensity, enabling droplets to attain greater transport velocity and broader spatial coverage. Simultaneously, shear-induced breakup reduces the Sauter mean diameter (SMD) and increases droplet number concentration. Together, these effects amplify relative velocity, effective cross section, and collision/adhesion efficiency. Consequently, at the same distance, a higher *Q*_g_ invariably yields greater η and approaches saturation more rapidly. Under conditions of *Q*_g_ ≤ 50 L/min, however, even at *L* ≈ 5 m, high suppression efficiency remains unattainable.
(2) At a fixed airflow rate, the liquid flow rate lies within the range of *Q*_l_ = 5–30 L/h; the process is predominantly governed by the number of droplets. Within this interval, the efficiency curves likewise shift upward as *Q*_l_ increases, with *L*_50_ and *L*_90_ sequentially shortened, following the order *Q*_l_ = 30 > 25 > 20 > 15 > 10 > 5 L/h. The governing mechanism lies in the increase of droplet number flux (*n*_d_ ∝ *Q*_l_/*D*_w_ ^3^). Within this parameter range, the enhancement of the capture probability due to the growing droplet population outweighs the adverse effects associated with larger droplet sizes, such as reduced flowability and premature sedimentation. As a result, the overall efficiency exhibits continuous improvement without any observable decline.
(3) When the liquid flow rate is *Q*_l_ = 7.2 L/h and the airflow rate is *Q*_g_ = 200 L/min, achieving a dust suppression efficiency above 80% requires an effective spray distance exceeding 3 m. As the airflow rate increases, the distance required for droplets to maintain effective action decreases. Specifically, when *Q*_g_ = 150 L/min and the effective spray distance surpasses 4 m, the suppression efficiency readily exceeds 80%.

## 3 Experimental Investigation of Ultrasonic Atomization Flow Characteristics

### 3.1 Construction of the Ultrasonic Atomization Experimental Platform

To quantitatively characterize the atomization behavior of a swirl-type ultrasonic nozzle and ensure repeatability and controllability of the experiments, the Dust Control Laboratory at the University of Science and Technology Beijing established an ultrasonic atomization experimental platform, composed of three subsystems: the gas circuit, the water circuit, and the measurement apparatus, as illustrated in Figure 6. This platform enables systematic testing of inlet gas–liquid parameters (pressure and flow rate) as well as outlet atomization characteristics (spray angle and effective range).

**Figure 6:**
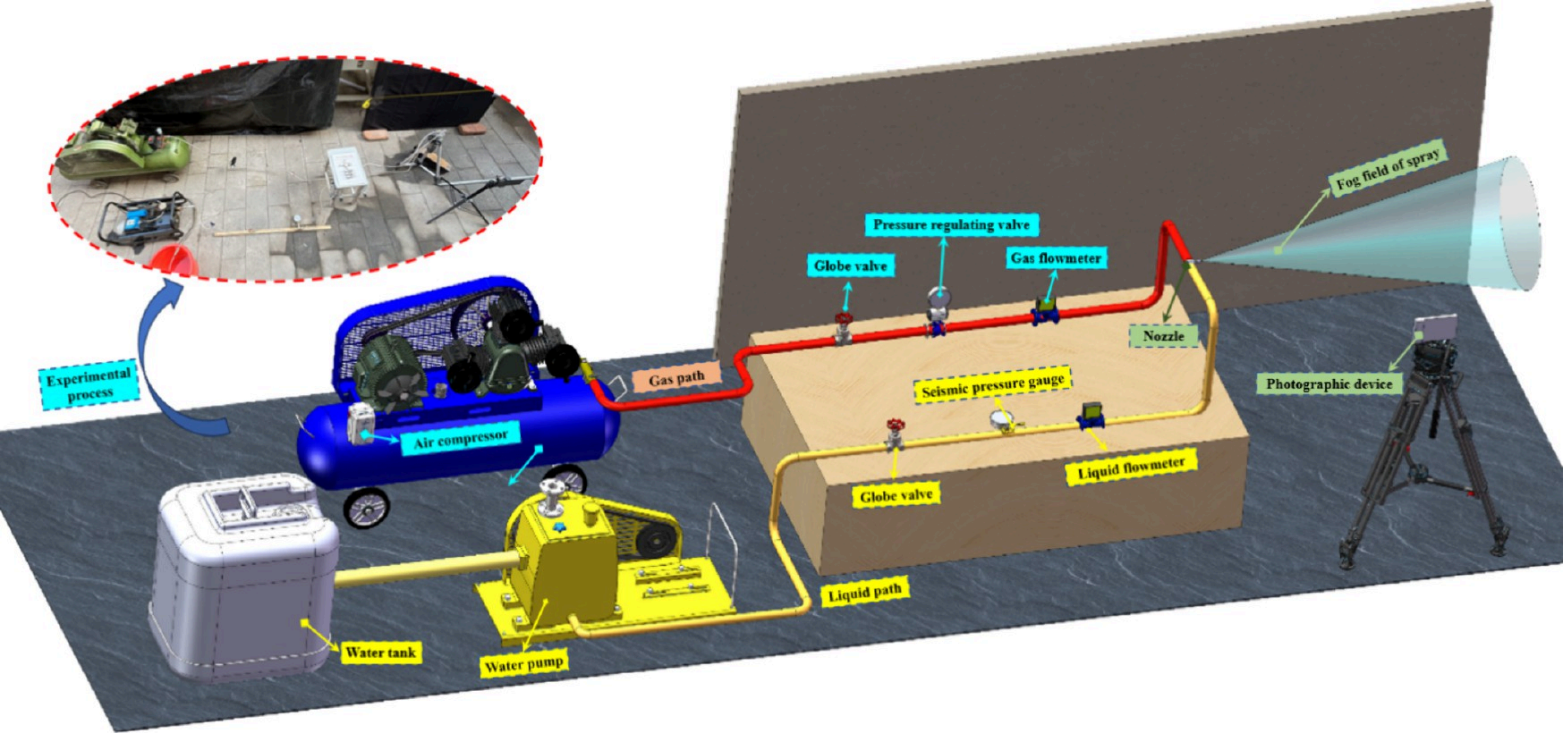
Schematic diagram of the atomization experimental device.

The gas circuit consists of a compressor, a shut-off valve, a pressure-regulating valve, and a gas flowmeter. The gas source is supplied by the compressor, with a pressure range of 0.1–0.8 MPa. The shut-off valve regulates the on/off state of the circuit, while the pressure-regulating valve allows fine adjustment of gas pressure within 0–1.0 MPa, with a precision of 0.01 MPa. A rotameter displays the gas flow rate.

The water circuit comprises a water tank, a pump, a shut-off valve, a pressure stabilizer, and a liquid flowmeter. Water pressure is provided by a QL-380A cleaning pump, adjustable within 0–2 MPa. The pressure stabilizer ensures a steady water supply, while the flowmeter records the liquid flow rate.

The measurement apparatus primarily employs a photographic system in conjunction with a calibrated ruler to determine the spray angle and the effective range of the nozzle.

To ensure the reliability of the experimental data, the accuracy levels of the instrumentation were as follows: the gas pressure gauge (±0.25% F.S.), the liquid pressure gauge (±0.5% F.S.), the gas flowmeter (±1.0%), and the liquid flowmeter (±1.5%). Each operating condition was measured three times, and the average value was taken as the final reported result.

### 3.2 Influence of Air Pressure on Ultrasonic Atomization Characteristics

Figure 7 presents the measured effects of air pressure on the atomization behavior of the swirl-type ultrasonic nozzle under varying liquid pressure conditions. The analysis shows that as air pressure increases from 0.15 to 0.75 MPa, gas flow rises markedly, while liquid flow exhibits an inverse declining trend. Correspondingly, both the spray angle and effective spray range increase with rising air pressure, with the low-pressure region (0.1–0.4 MPa) demonstrating the highest sensitivity and the high-pressure region (>0.6 MPa) gradually approaching saturation. At an air pressure of 0.15 MPa and a liquid pressure of 0.5 MPa, the minimum values are observed: a gas flow of 25 L/min, a spray angle of 14°, and an effective range of 122 cm. Conversely, at an air pressure of 0.75 MPa and a liquid pressure of 0.1 MPa, the maximum values are recorded: a gas flow of 136 L/min, a spray angle of 57°, and an effective range of 281 cm. These results indicate that the gas–liquid ratio is the pivotal factor driving the evolution of atomization performance. With increasing air pressure, gas-phase momentum intensifies rapidly, generating strong swirling motion and high-velocity shear fields within the nozzle. Under such conditions, the liquid jet is more readily stripped and fragmented into fine droplets, leading to a reduction in liquid flow.^29^ Simultaneously, the smaller droplet size significantly enhances spray diffusion and spatial coverage, while the reduction in droplet inertia extends the effective range.

**Figure 7:**
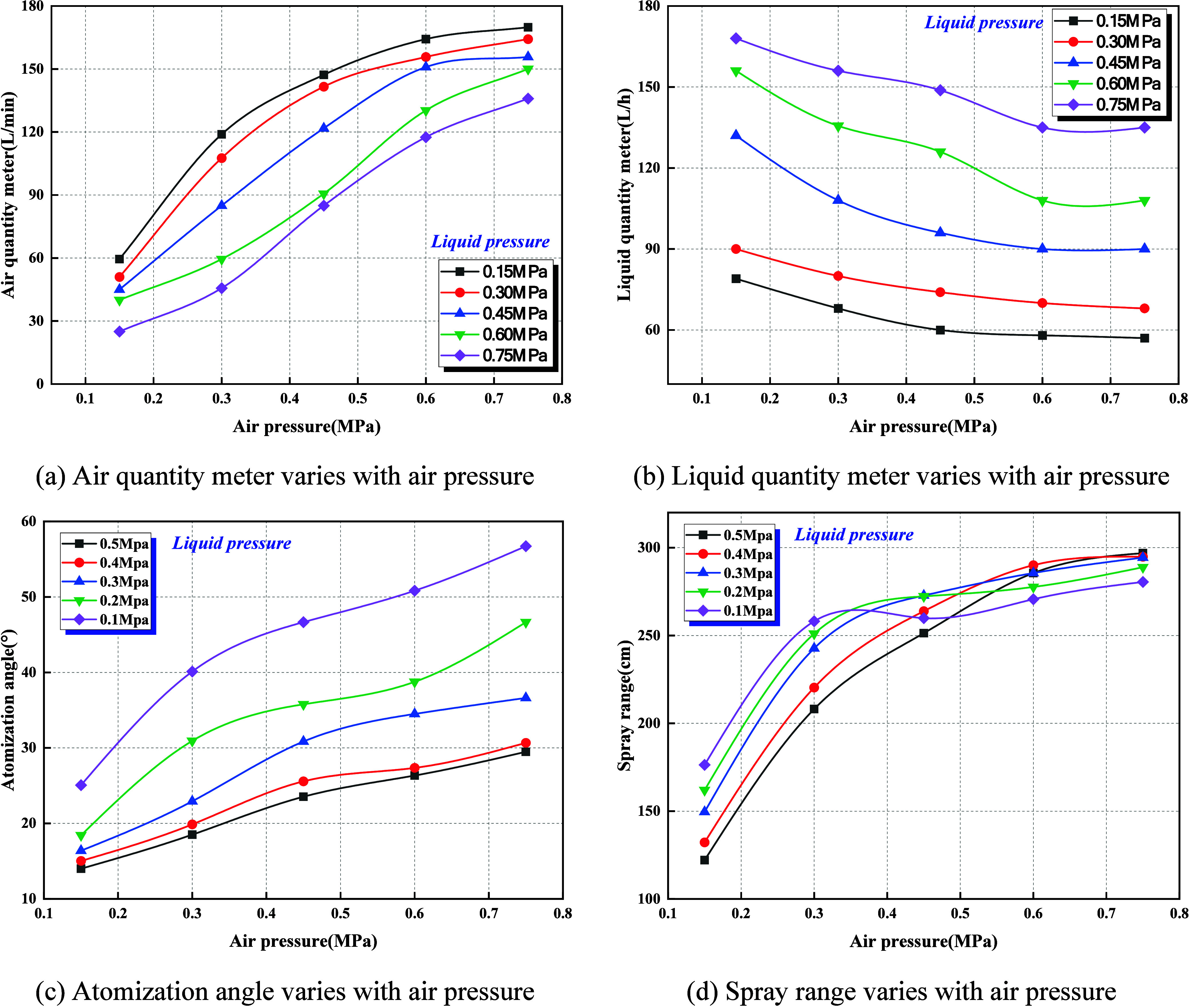
Effect of air pressure on ultrasonic atomization characteristics under different liquid pressure conditions. (a) Air quantity meter varies with air pressure. (b) Liquid quantity meter varies with air pressure. (c) Atomization angle varies with air pressure. (d) Spray range varies with air pressure.

### 3.3 Influence of Liquid Pressure on Ultrasonic Atomization Characteristics

From Figure 8a,b, it is evident that gas flow decreases significantly as liquid pressure rises from 0.1 to 0.5 MPa, with the reduction becoming less pronounced at higher air pressures: at 0.75 MPa, the decline is about 20%, whereas at 0.15 MPa, it reaches nearly 58%. During this process, liquid flow increases monotonically with liquid pressure, reaching its minimum at 0.1 MPa and its maximum at 0.5 MPa. These results indicate that increasing liquid pressure alters gas–liquid momentum distribution and reduces the gas–liquid ratio (*Q*_g_/*Q*_l_), thereby imposing a channel-occupying effect on the gas phase at the inlet, which diminishes effective gas transport capacity.

**Figure 8:**
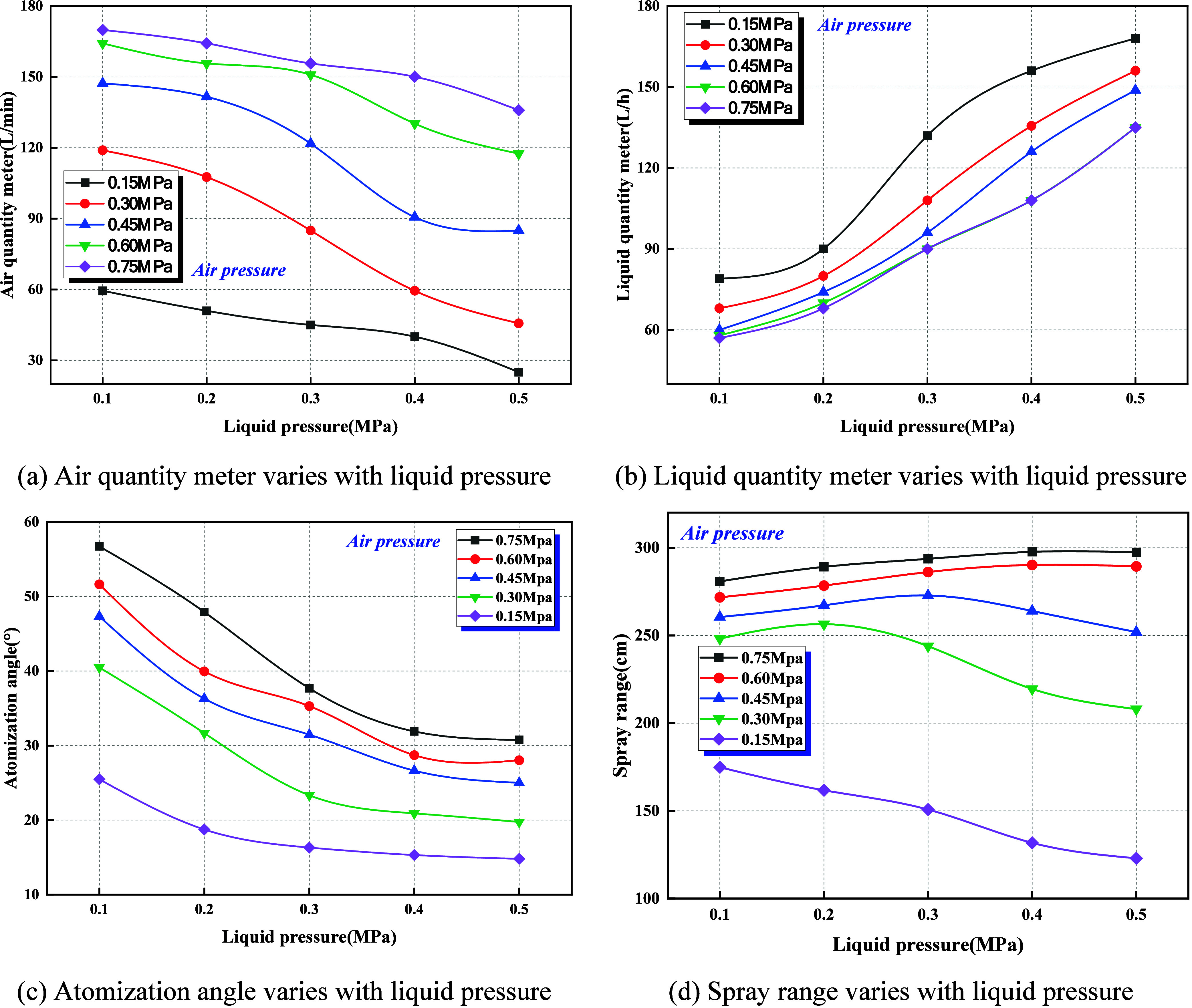
Effect of liquid pressure on ultrasonic atomization characteristics under different air pressure conditions. (a) Air quantity meter varies with liquid pressure. (b) Liquid quantity meter varies with liquid pressure. (c) Atomization angle varies with liquid pressure. (d) Spray range varies with liquid pressure.

Figure 8c,d further shows that the spray angle and effective range contract with rising liquid pressure. At low liquid pressure and relatively high air pressure, the spray angle can reach 50–60°, but it shrinks to below 20° when liquid pressure increases to 0.5 MPa. Similarly, the effective range can exceed 280 cm at an air pressure of 0.75 MPa, yet it falls to less than 150 cm under high liquid pressure. This suggests that enhanced liquid supply suppresses gas–liquid shear and droplet dispersion induced by spray field perturbations, manifesting macroscopically as a reduced spray range and spatial coverage.

Thus, elevated liquid pressure exerts a systemic influence on the atomization process by altering the gas-to-liquid ratio (*Q*_g_/*Q*_l_). On the one hand, it improves liquid supply stability and flow rate; on the other hand, it increases droplet breakup resistance, lowers secondary atomization efficiency, and consequently leads to contraction of the spray angle and shortening of the effective range. This pattern reflects the intrinsic mechanism of gas–liquid momentum competition: when the liquid-phase momentum dominates, droplet cohesion and surface tension effects prevail, counteracting the dispersive forces of swirling flow and ultrasonic oscillation.^30^ Notably, this tendency is especially pronounced under high air pressure, underscoring the existence of a complex nonlinear coupling between liquid pressure and air pressure, where elevated liquid pressure not only hinders gas-phase momentum transfer but also weakens the droplet refinement effect of ultrasonic atomization, thereby reducing the overall atomization performance of the nozzle.

## 4 Numerical Simulation of Ultrasonic Atomization Flow Field Characteristics

### 4.1 Geometric Model Establishment and Grid Division

To quantitatively elucidate the influence of air pressure, liquid pressure, gas–liquid ratio, and surface tension on spray velocity, droplet size, and droplet concentration in swirl-type ultrasonic atomization, this study employs the discrete phase model (DPM) in ANSYS Fluent. At the nozzle outlet, an engineering-scale initial droplet population is generated using the air-blast/air-assist injection model, without resolving the internal nozzle flow.^12,31^ The injection model incorporates an equivalent nozzle diameter, coaxial gas channel geometry, gas/liquid mass flow rate (or inlet pressure drop), and spray angle as inputs. These parameters, calibrated by the experiments described in Section 4.2, establish the correspondence between *Q*_g_/*Q*_l_ and the initial droplet spectrum. The continuous phase is solved with a pressure-based transient RANS approach (realizable *k–*ε model with enhanced wall treatment), where air is treated as a compressible ideal gas with the energy equation and gravity enabled. The discrete and continuous phases are two-way coupled, evaporation is disabled (dry-fog condition), secondary breakup is modeled using the KH–RT approach, and droplet–droplet collisions are handled via the O’Rourke algorithm.^27,32^ Boundary conditions are aligned with experimental ranges: the air inlet as pressure-inlet (gauge), *P*_g_ = 0.15–0.75 MPa; the liquid inlet as pressure-inlet (gauge), *P*_l_ = 0.10–0.50 MPa; the outlet as pressure-outlet (0 Pa gauge); and walls as no-slip boundaries. The computational domain is three-dimensional, covering the maximum spray extent (axial ≥ 4.0 m, radial ≥ 2.0 m). Near the nozzle exit, grid refinement and strict time-step control are applied, with Δ*t* ≈ 2 μs to ensure local CFL < 0.25 for numerical stability. To characterize the modulation of atomization by fluid properties, surfactant solutions with mass fractions of 0.0005%, 0.005%, 0.05%, 0.10%, and 0.50% were prepared according to the WANG formulation, as shown in Figure 9a. Their surface tension (σ) was measured by using a tensiometer and incorporated into the breakup criterion and Weber number calculation. In this way, the response of the velocity field, SMD/MVD, and droplet volume fraction was systematically evaluated under various combinations of *P*_g_, *P*_l_, *Q*_g_/*Q*_l_, and σ. The mesh division is refined along the symmetry axis, and the average cell mass of the grid is very large, with the maximum mesh mass of 1, the minimum mesh mass of 0.0186, the average mass of 0.8964, and the average skewness of 0.0818, as shown in Figure 9b, which meets the requirements of calculation accuracy.

**Figure 9:**
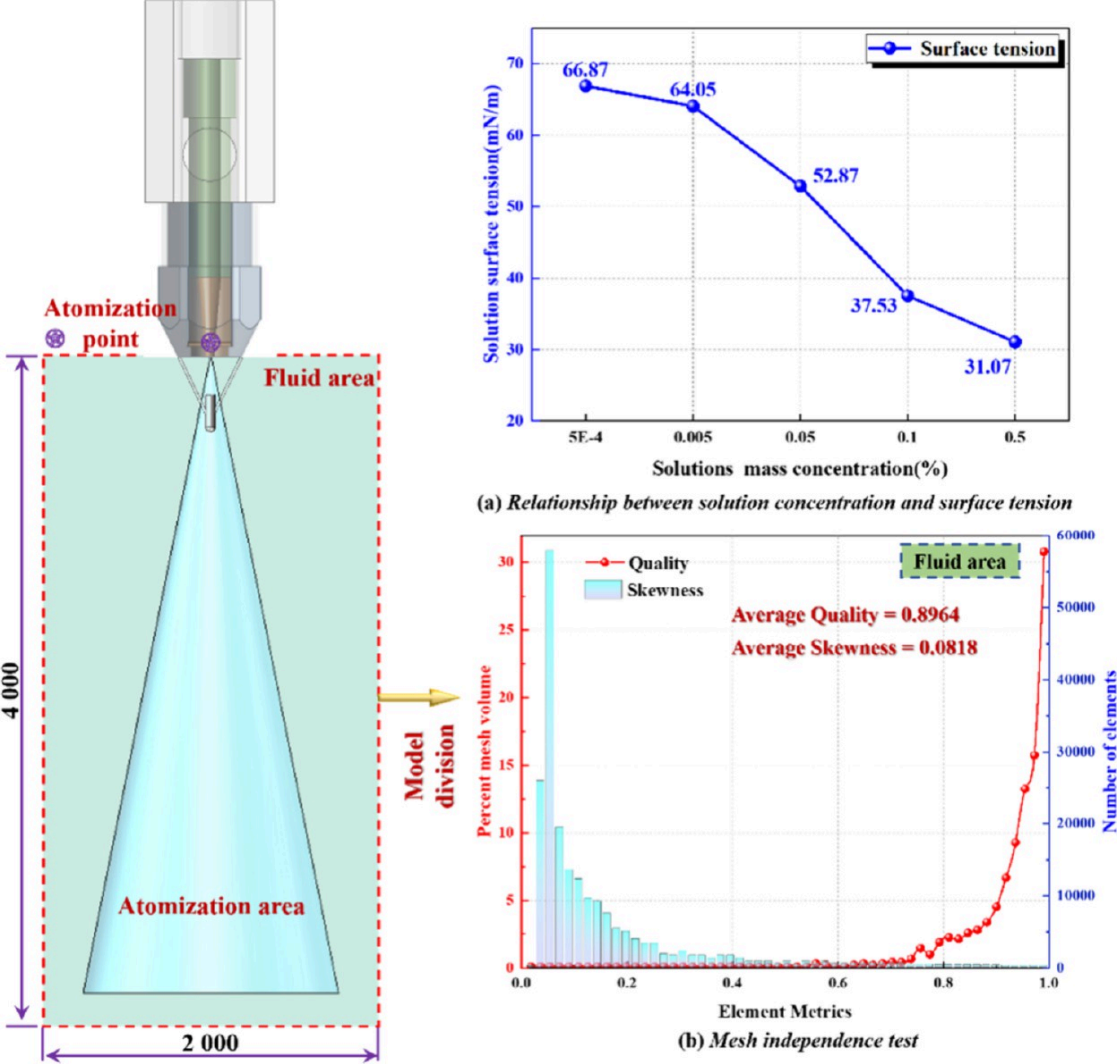
(a, b) Physical model and mesh division.

### 4.2 Comparison and Validation of Simulation with Experimental Results

Figure 10 depicts the temporal evolution of the spray within 5–300 μs after injection. The droplet cloud advances axially from a dense near-field cone to approximately 3.0–3.5 m, while the outer cone angle remains essentially stable. Stacked-bar analyses quantitatively reveal the monotonic shift of the droplet spectrum toward smaller sizes over time. Specifically, the fraction of large droplets (80–100 μm) decreases sharply, from 28% at 5 μs to 23% at 10 μs, 8% at 100 μs, and only 3% at 300 μs. Conversely, the proportion of fine droplets (0–30 μm) rises steadily, from 3–5% at 5–10 μs to 16% at 150 μs, 20% at 200 μs, and 22% at 300 μs. Droplets in the 30–40 μm range likewise increase from 4–7% to 15–19% within the same interval.

**Figure 10:**
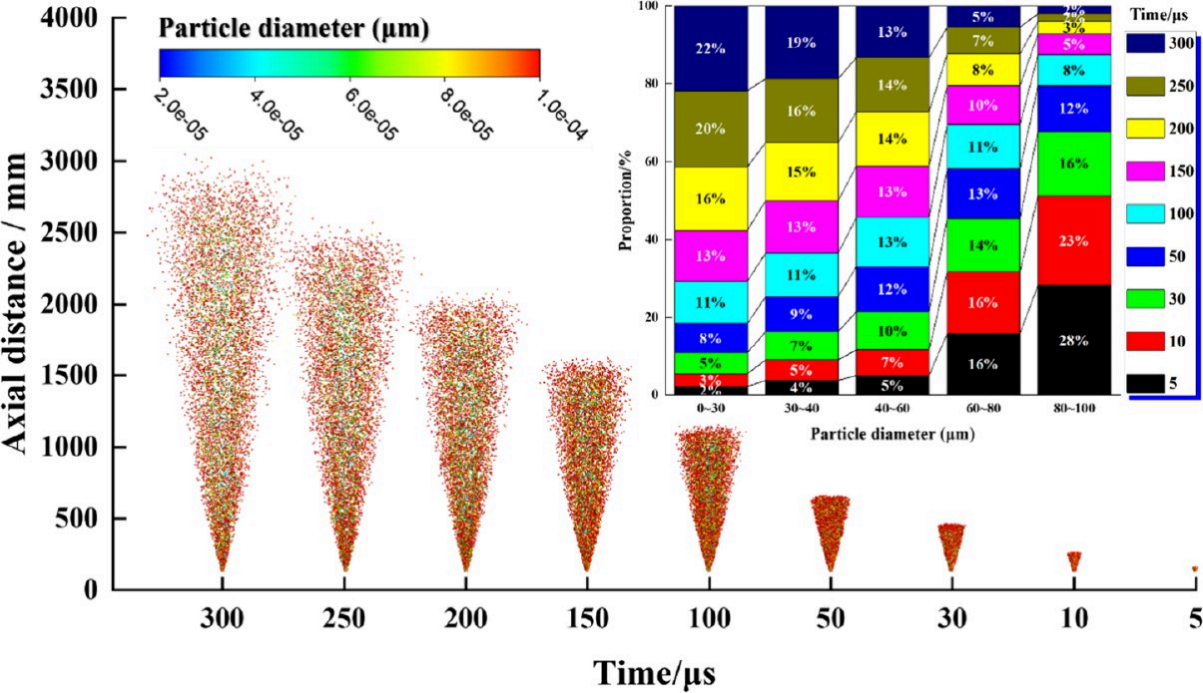
Variation of droplet size with time and its proportion.

Overall, the droplet spectrum exhibits a clear temporal migration toward smaller sizes. The spray evolution can be divided into two stages: an initial “rapid redistribution period” (0–150 μs), during which the proportion of large droplets declines precipitously while that of fine droplets rises sharply, followed by a “gradual stabilization period”, in which both the spectrum and cone angle approach steady values, though the cloud continues to extend outward. Concurrently, the axial advancement of the spray grows nearly linearly, while the droplet cloud gradually dilutes. This behavior arises from the combined effects of secondary breakup and size-selective transport under high relative velocity conditions. Elevated Weber numbers trigger KH–RT instabilities that cause the rapid erosion and fragmentation of large droplets into medium and fine droplets. As the droplet size decreases, both Reynolds and Stokes numbers decline, rendering smaller droplets more susceptible to entrainment and diffusion with the primary airflow, thereby increasing their proportion over time. Larger droplets, by contrast, deviate more readily from the mainstream or undergo continued breakup due to inertia and gravity, leading to a persistent reduction in their fraction. This spatiotemporal pattern of “large-to-small migration and near-to-far dispersion” aligns well with air-blast atomization theory and DPM–KH–RT model predictions.

Figure 11 illustrates the spatiotemporal evolution of the droplet velocity field within 5–300 μs after spray initiation. The velocity distribution reveals that the axial penetration distance extends to approximately 3.5–4.0 m, while the outer conical boundary remains nearly unchanged. At the far-field boundary, droplets have undergone substantial deceleration, entrainment, dispersion, and gravitational settling, resulting in very low residual velocities. The stacked histogram further quantifies this shift: the proportion of the low-velocity fraction (0–10 mm/s) decreases from 17% at 5 μs to 7% at 300 μs, whereas the fractions within 20–30, 30–40, and 40–50 mm/s progressively rise from 8% to 17%, 3% to 18%, and 3% to 22%, respectively; meanwhile, the 10–20 mm/s group remains nearly constant at 10–12%. These results indicate, first, that a pronounced “migration toward higher velocities” occurs within 0–150 μs, followed by a quasi-steady distribution dominated by the 20–50 mm/s range; and second, that the rightward shift of the distribution coincides with the linear extension of axial penetration, signifying that flow field modulation is governed primarily by convective transport rather than geometric expansion. This behavior arises because droplets, subjected to high-speed airflow, relax toward the convective velocity according to d*v*/d*t* ∝ (*u*_g_ – *v*)/τ_p_, where the particle response time τ_p_ ∼ ρ_l_ *d* ^2^/(18*u*_g_) diminishes with a decreasing droplet diameter. Combined with droplet spectrum refinement induced by secondary breakup, the reductions in τ_p_ and Stokes numbers strengthen gas–droplet coupling, thereby driving the velocity distribution rightward and favoring dominance in the intermediate-to-high velocity regimes.

**Figure 11:**
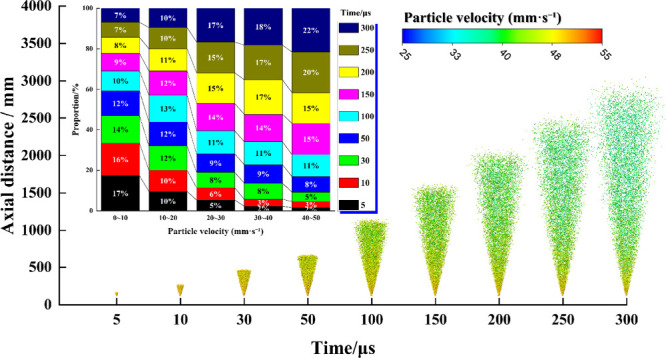
Variation of droplet velocity with time and its proportion.

Figure 12 presents a comparative analysis between the visualized experimental photographs (left) and the projected droplet-density fields derived from numerical simulation (right) across the operating matrix of “gas pressure (columns) × liquid pressure (rows)”. Overall, the two exhibit strong consistency in both the monotonic trends and the relative magnitudes of spray morphology under varying conditions. At fixed liquid pressure, increasing gas pressure from 0.15 to 0.75 MPa transforms the spray from a “slender jet–narrow cone” into a “diffuse cloud–wide cone”, characterized by a pronounced expansion of the outer cone angle, enlargement of the peripheral diffusion zone, and extended penetration distance. Conversely, at fixed gas pressure, raising the liquid pressure from 0.10 to 0.50 MPa induces the opposite tendency: the spray narrows, the axial dense core elongates, and the system exhibits a stronger “jet-like” character.

**Figure 12:**
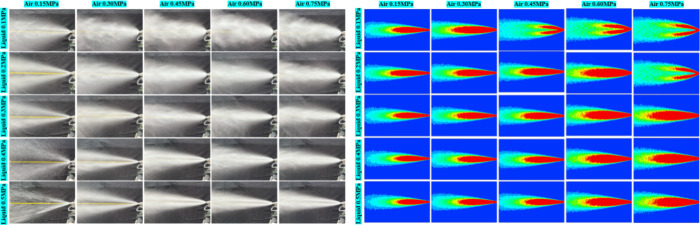
Comparison and verification between simulation results and experimental results.

These phenomena can be unified under a governing law dictated by the ratio *Q*_g_/*Q*_l_. An increase in *Q*_g_/*Q*_l_ enhances gas-phase momentum and shear, thereby widening the atomization angle and lengthening the spray range; in contrast, a decrease in *Q*_g_/*Q*_l_ strengthens the jet continuity and cohesion, coarsening the droplet size, contracting the cone angle, and elongating the near-field core. In the simulations, the radial transition from red to cyan in the isopleths corresponds closely to the brightness attenuation zones observed in the experimental images. Furthermore, at elevated gas pressures (≥0.60 MPa), the rate of outward expansion tends toward saturation, reflecting diminishing atomization gains, which is consistent with the conclusions drawn from the parametric mapping.

### 4.3 Analysis of Influencing Factors of Spray Fog Field Velocity

Using CFD-Post, the outlet cross section was extracted to evaluate the mean droplet velocity under varying gas and liquid pressures, as illustrated in Figure 13. The outlet cross section refers to the outflow boundary located 4.0 m downstream of the nozzle rather than the physical nozzle exit.

**Figure 13:**
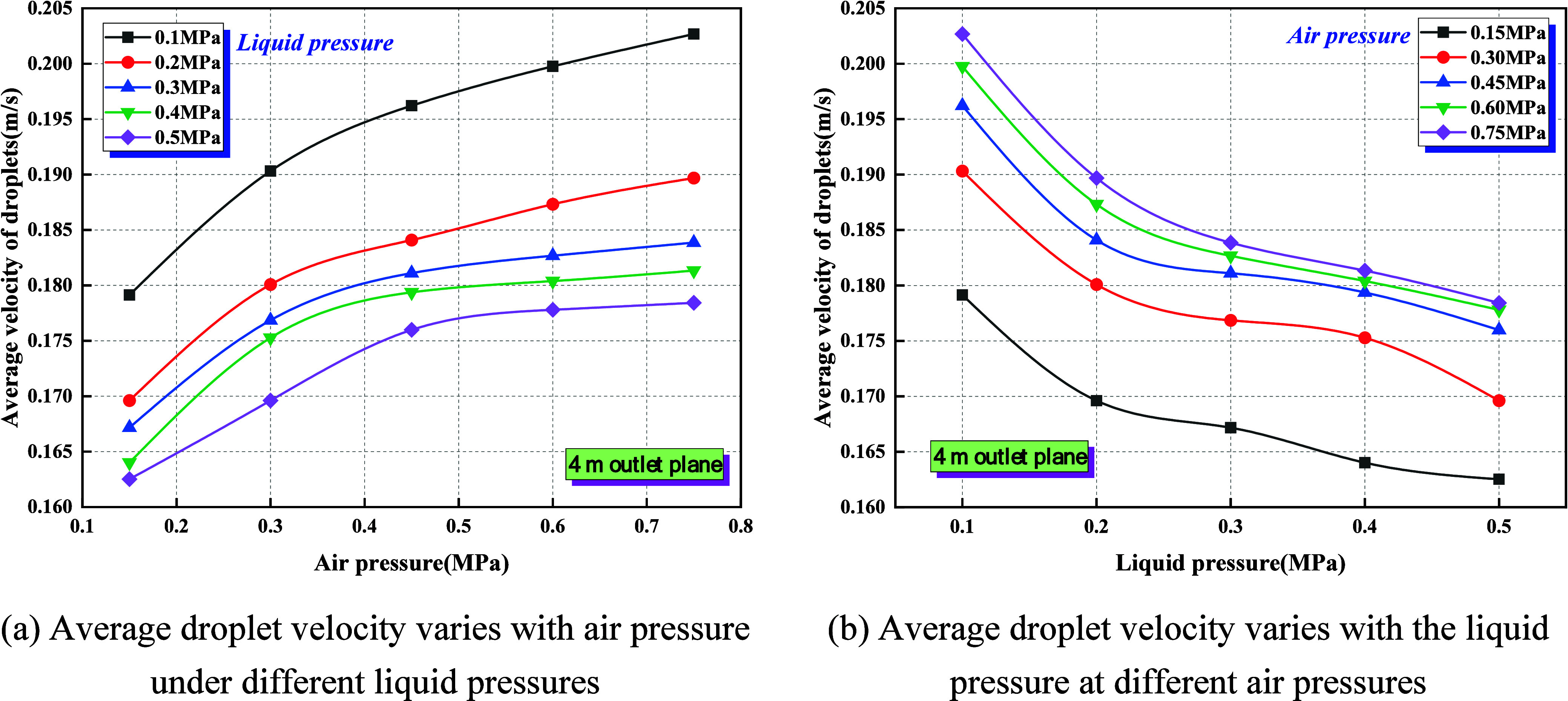
Effect of gas–liquid pressure on average velocity of droplets. (a) Average droplet velocity varies with air pressure under different liquid pressures. (b) Average droplet velocity varies with the liquid pressure at different air pressures.

(1) As shown in Figure 13a, under fixed liquid pressure, the mean droplet velocity rises monotonically with increasing gas pressure from 0.15 to 0.75 MPa, with the most pronounced growth occurring in the low-pressure regime (0.15–0.30 MPa), after which the curve gradually levels off into a plateau (≥0.60 MPa). In this process, when *P*_l_ = 0.10 MPa, the mean velocity increases from approximately 0.179 to 0.203 m/s, a 13% rise; whereas at *P*_l_ = 0.50 MPa, it grows only from 0.163 to 0.179 m/s, representing a 10% increase. Figure 13b shows that under fixed gas pressure, the mean droplet velocity decreases monotonically as liquid pressure rises from 0.10 to 0.50 MPa, with the steepest decline occurring in the low-pressure range (0.10–0.20 MPa), followed by a gradual attenuation. In this process, at *P*_g_ = 0.75 MPa, the mean velocity falls from 0.202 to 0.178 m/s (12% reduction), while at *P*_g_ = 0.15 MPa, it drops from 0.179 to 0.163 m/s (9% reduction).
(2) An increase in *P*_g_, or equivalently in *Q*_g_/*Q*_l_, elevates the mean droplet velocity; conversely, an increase in *P*_l_, or a reduction in *Q*_g_/*Q*_l_, lowers it. Higher gas pressure enhances gas-phase momentum and entrainment, refining droplet size, thereby reducing particle response time (τ_p_ ∝ *d* ^2^) and the Stokes number; the gas–droplet slip diminishes, enabling droplets to more rapidly relax toward the convective air velocity. In contrast, increasing liquid pressure augments liquid-phase momentum and jet cohesion, suppressing primary and secondary breakup, enlarging droplet size, lengthening τ_p_, weakening coupling, and thus depressing the mean velocity. The plateau behavior observed at elevated gas pressure reflects the asymptotic limit imposed by the mainstream convective velocity, whereas the convergence of curves at high liquid pressure reveals nonlinear coupling under liquid inertia dominance and diminishing marginal effects.

As shown in Figure 14, both the liquid–gas flow ratio (*Q*_l_/*Q*_g_) and the droplet velocity (*v*) exhibit power-law relationships with the liquid–gas pressure ratio (*P*_l_/*P*_g_), expressed as *Q*_l_/*Q*_g_ = 6.16­(*P*_l_/*P*_g_)^−1.4^ and *v* = 0.176­(*P*_l_/*P*_g_)^−0.067^, with correlation coefficients *R* ^2^ of 0.953 and 0.973, respectively. The absolute value of the power exponent for *Q*_l_/*Q*_g_ is far greater than that for droplet velocity, indicating that the pressure ratio exerts a pronounced influence on the gas–liquid flow share while exerting only a weak dependence on the velocity field, which trends toward an asymptotic plateau. Specifically, when *P*_l_/*P*_g_ increases from 0.5 to 1.5, *Q*_l_/*Q*_g_ decreases by approximately 3^1.4^ ≈ 4.7, whereas *v* decreases by only about 3^0.067^ ≈ 10%.

**Figure 14:**
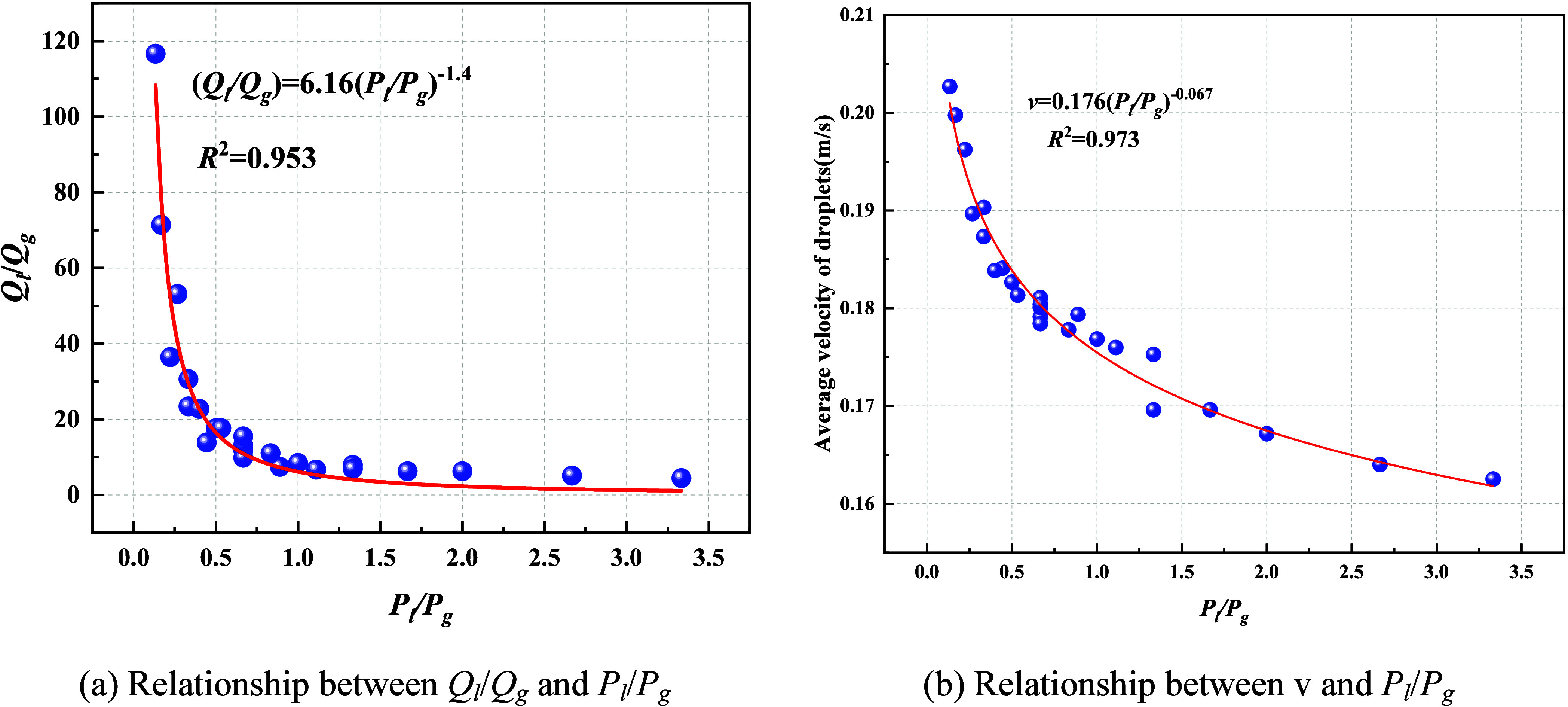
Relationship between *Q*_l_/*Q*_g_, *v*, and *P*_l_/*P*_g_. (a) Relationship between *Q*_l_/*Q*_g_ and *P*_l_/*P*_g_. (b) Relationship between *v* and *P*_l_/*P*_g_.

These results suggest that in scenarios where reducing liquid consumption or expanding spray coverage is desired while largely maintaining transport capacity, *P*_l_/*P*_g_ can be moderately increased to sharply suppress *Q*_l_/*Q*_g_ with only minor effects on *v*. Conversely, when the objective is to strengthen droplet momentum and dust suppression efficacy, a lower *P*_l_/*P*_g_ (achieved by raising *P*_g_ or lowering *P*_l_) should be maintained to enhance aerodynamic shear, refine the droplet spectrum, and elevate mean velocity.

### 4.4 Analysis of Influencing Factors of Spray Droplet Size

Figure 15 systematically characterizes the coupled influence of gas pressure (*P*_g_) and liquid pressure (*P*_l_) on the atomization scale based on CFD-Post statistics of the nozzle outlet cross section.

**Figure 15:**
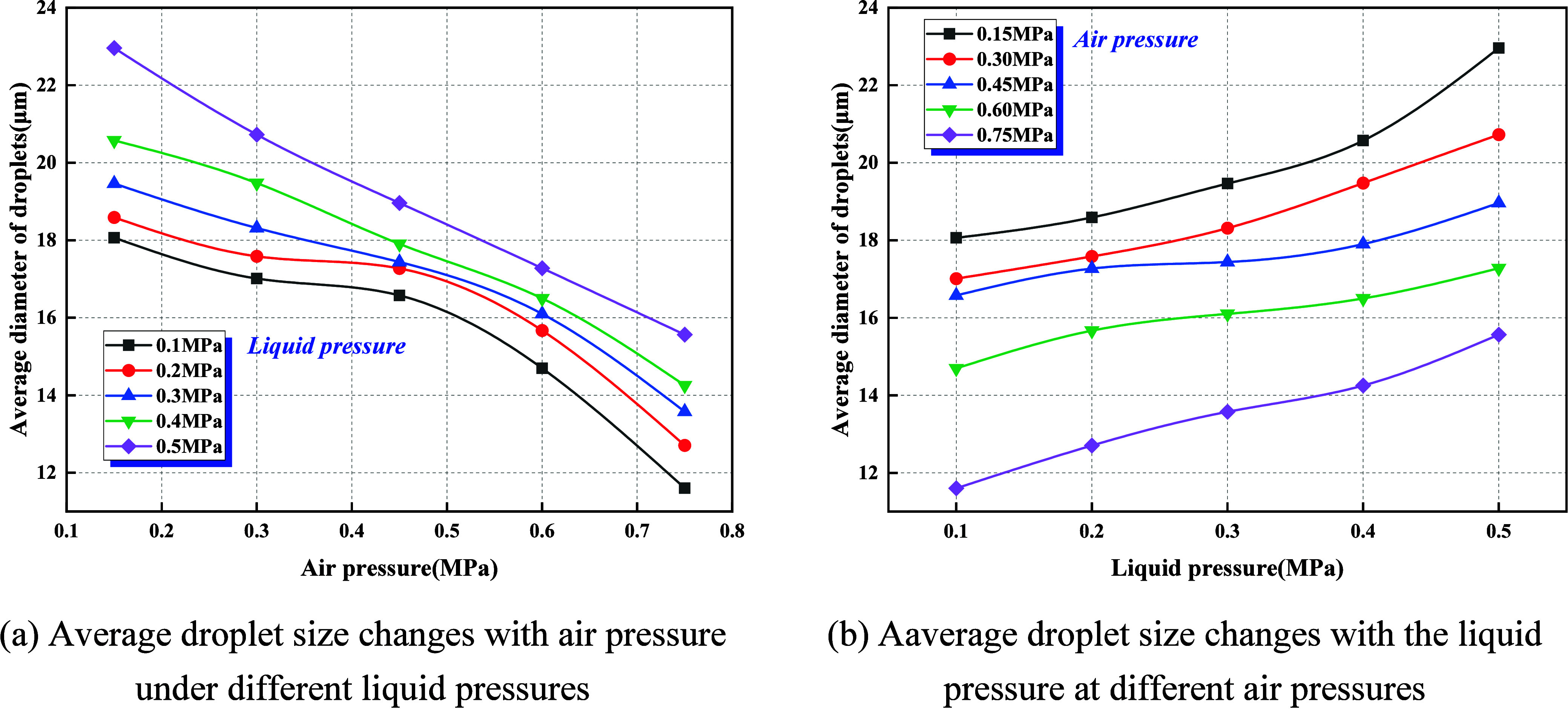
Effect of gas–liquid pressure on average droplet size. (a) Average droplet size changes with air pressure under different liquid pressures. (b) Average droplet size changes with the liquid pressure at different air pressures.

(1) As shown in Figure 15a, at fixed *P*_l_, the mean droplet size decreases nearly linearly with increasing *P*_g_. When *P*_l_ rises from 0.1 to 0.5 MPa, the mean diameter declines from approximately 18.5–22.5 μm at *P*_g_ = 0.15 MPa to 11.5–15.5 μm at *P*_g_ = 0.75 MPa, corresponding to a reduction of 25–40%. Figure 15b demonstrates that at fixed *P*_g_, the mean droplet size increases monotonically with *P*_l_. At *P*_g_ = 0.15 MPa, the droplet size expands from 18 μm at *P*_l_ = 0.1 MPa to 23 μm at *P*_l_ = 0.5 MPa (28% increase), while at *P*_g_ = 0.75 MPa, the corresponding growth converges to 25%.
(2) Raising *P*_g_ increases gas–liquid relative velocity and gas density, thereby elevating the gas-phase Weber number (*We*) and the gas-to-liquid momentum ratio (*Q*_g_/*Q*_l_). This drives the liquid jet or film to transition from wave–bag breakup toward strip detachment, intensifying both primary and secondary fragmentation and thus reducing mean droplet size. In contrast, elevating *P*_l_ augments the liquid flow rate and jet thickness, enhancing the competition of liquid inertia and viscosity against surface tension, prolonging breakup length, and enlarging the initial droplet size.
(3) From an engineering perspective, for the capture of respirable dust (1–10 μm), high-gas-pressure and low-liquid-pressure conditions (*P*_g_ ≥ 0.6 MPa, *P*_l_ ≤ 0.2 MPa) are optimal, yielding droplet sizes concentrated in the 12–15 μm range with strong secondary breakup potential. For total dust suppression, however, *P*_l_ may be moderately increased (0.4–0.5 MPa) to enhance liquid flux and spatial coverage.

Under conditions of liquid pressure at 0.1 MPa and gas pressure at 0.45 MPa, the spatial distribution of the droplet size under varying liquid concentrations is compared, as shown in Figure 16. Changes in concentration do not markedly alter the axial or radial distribution patterns: axially, droplet size increases with the distance from the nozzle, while radially, droplet size exhibits a symmetric profile about *Y* = 0. With rising liquid concentration, the droplet size at all spatial sampling points shows an overall decreasing trend. The reduction is particularly pronounced as concentration increases from 0.005% to 0.5%, whereas the decline moderates when concentration rises from 0.4% to 0.5%, reflecting a saturation effect. This phenomenon suggests the existence of a threshold in the reduction of droplet size with increasing concentration. Once the activator concentration surpasses 0.4%, further decreases in droplet size become constrained, a behavior primarily attributable to saturation adsorption of activator molecules at the gas–liquid interface and to physical processes such as droplet coalescence.

**Figure 16:**
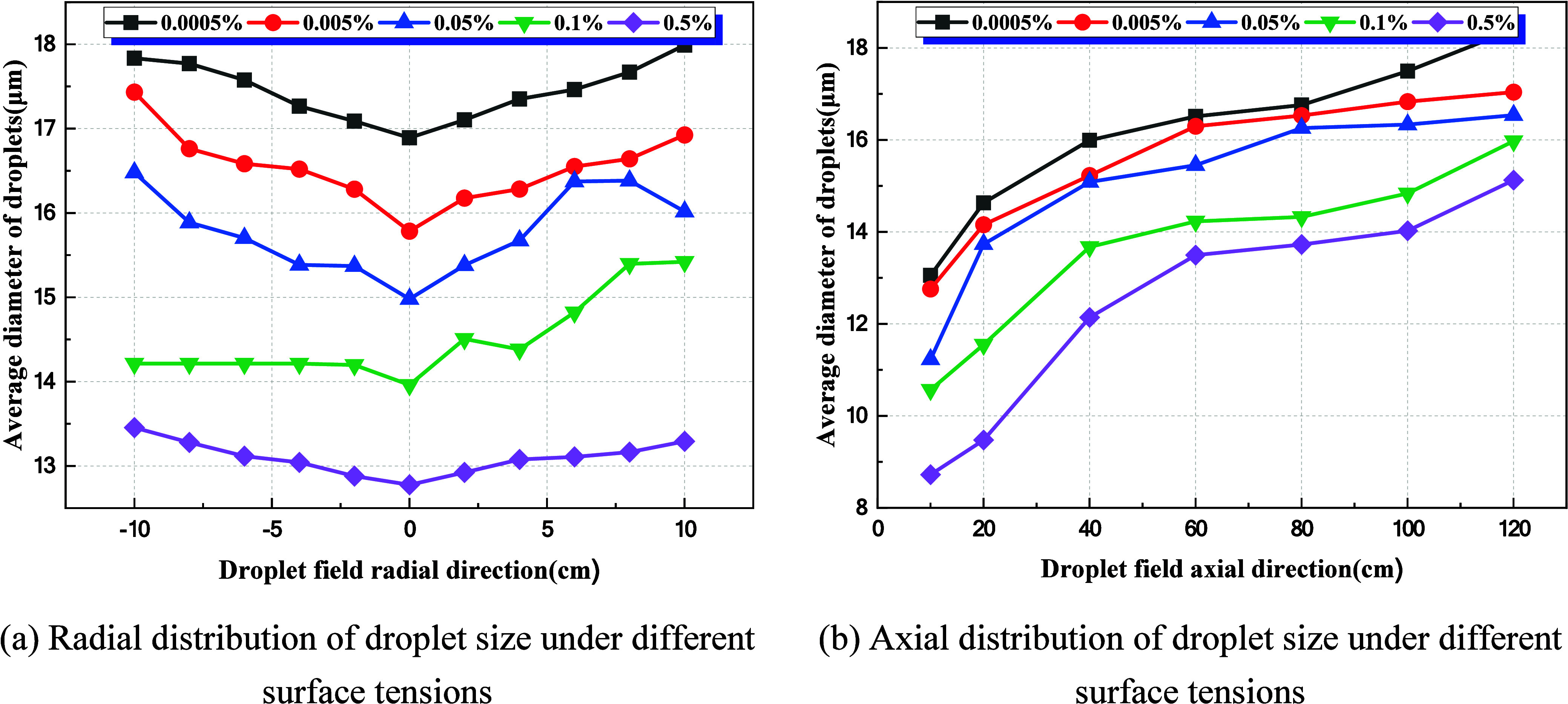
Effect of liquid surface tension on average droplet size. (a) Radial distribution of droplet size under different surface tensions. (b) Axial distribution of droplet size under different surface tensions.

### 4.5 Analysis of Influencing Factors of Spray Droplet Concentration

Figure 17 illustrates the spatial distribution of the droplet size under varying liquid pressures at a fixed gas pressure of 0.45 MPa. Figure 18, under a constant liquid pressure of 0.1 MPa, presents the corresponding distribution characteristics for different gas pressures.

**Figure 17:**
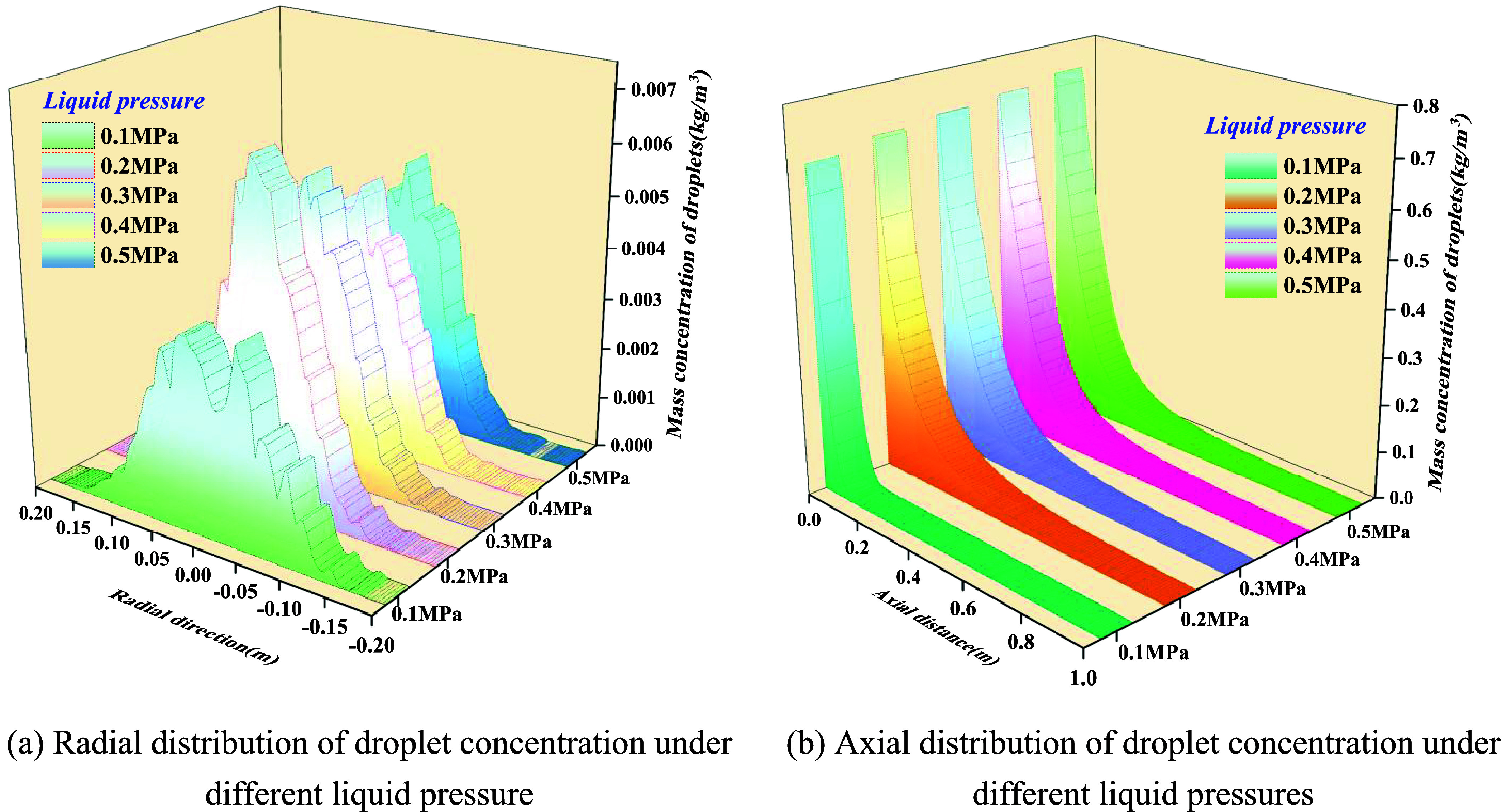
Effect of liquid pressure on droplet concentration. (a) Radial distribution of droplet concentration under different liquid pressures. (b) Axial distribution of droplet concentration under different liquid pressures.

**Figure 18:**
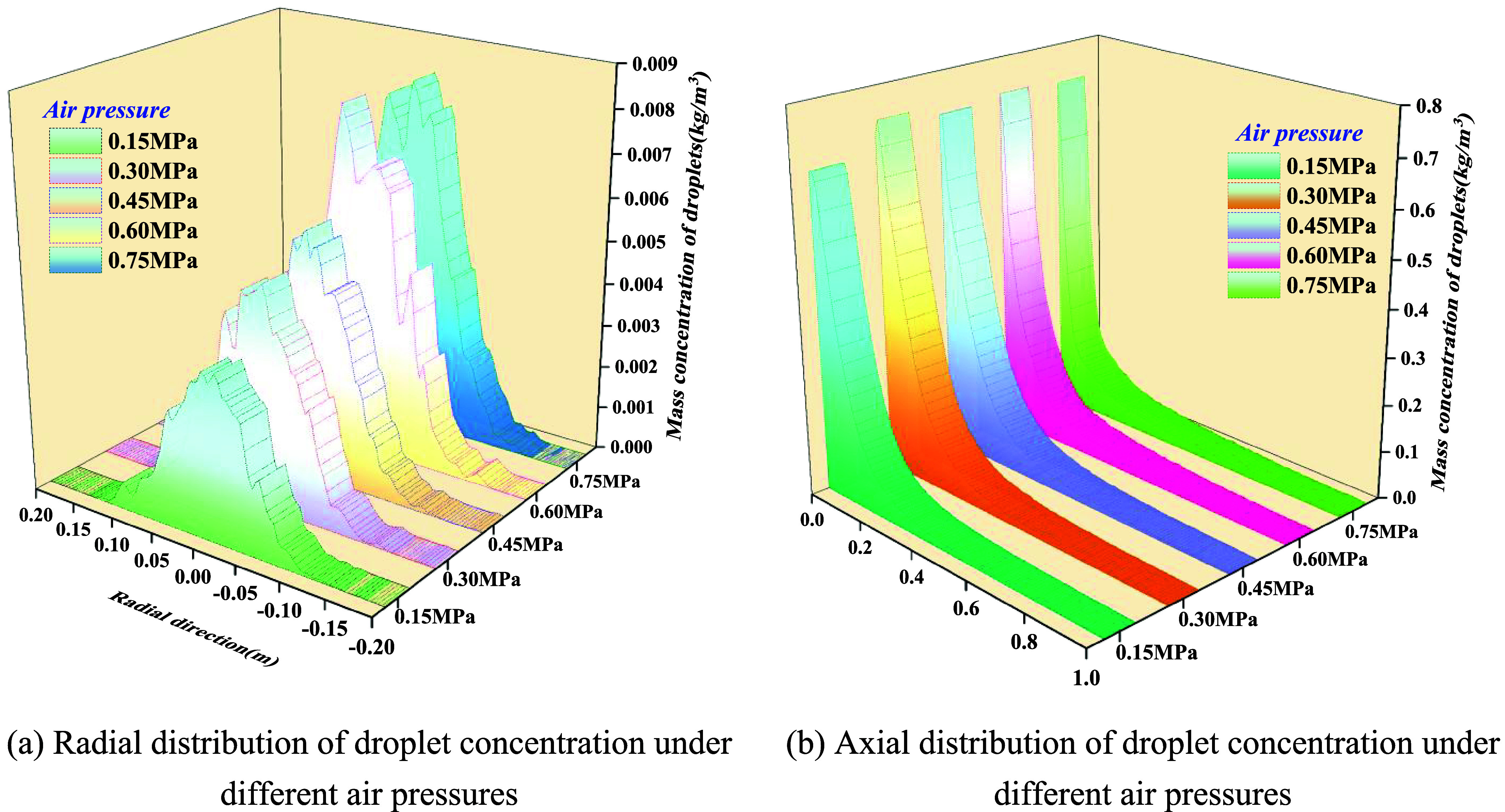
Effect of air pressure on droplet concentration. (a) Radial distribution of droplet concentration under different air pressures. (b) Axial distribution of droplet concentration under different air pressures.

(1) Radially, the droplet concentration exhibits a centrally symmetric, unimodal distribution, approximating a Gaussian profile. At fixed gas pressure, raising liquid pressure from 0.1 to 0.50 MPa simultaneously increases both the peak concentration and distribution width: the central peak rises from 3 × 10^–3^ to 6.5 × 10^–3^ kg/m^3^, while the half-width expands slightly, indicating that liquid pressurization delivers greater volumetric flux and drives the inertial outward spread of larger droplets. At fixed liquid pressure, increasing gas pressure from 0.15 to 0.75 MPa markedly elevates the central peak (from 4 × 10^–3^ to 9 × 10^–3^ kg/m^3^) while narrowing the radial spread, signifying that under strong aerodynamic shear, droplets are more firmly entrained by the high-speed core, leading to a more concentrated jet.
(2) Axially, the droplet concentration decays monotonically with the distance from the nozzle. Increasing either *P*_l_ or *P*_g_ elevates the overall profile while slightly extending the characteristic decay length. Near the nozzle exit, concentrations reach 0.4–0.8 × 10^–3^ kg/m^3^, with roughly 80% attenuation completed within 0.2–0.3 m, after which the field transitions into a low-concentration wake.
(3) Elevating gas pressure raises the Weber number (We) and momentum ratio, driving the liquid film from wave breakup toward strip detachment. This reduces droplet size, diminishes slip velocity, and intensifies convective entrainment, producing a regime of “higher axial concentration and narrower radial spread”. Conversely, increasing liquid pressure augments mass flux and jet thickness, generating larger droplets with stronger inertia, manifesting as “peak elevation with moderate radial broadening”. This behavior is reflected quantitatively as ∂*C*_0_/∂*P*_g_ > 0, ∂*C*_0_/*P*_l_ > 0, ∂σ_r_/*P*_g_ < 0, ∂σ_r_/*P*_l_ > 0, ∂λ/∂*P*_g_ > 0, and ∂λ/*P*_l_ > 0 (where *C*_0_ denotes centerline concentration, σ_r_ the radial half-width, and λ the axial decay length).
(4) For near-field, high-concentration, narrow-jet coverage aimed at rapid capture of respirable dust, a “high gas pressure–moderate liquid pressure” regime (*P*_g_ ≥ 0.6 MPa, *P*_l_ = 0.1–0.3 MPa) is recommended, yielding finer droplets and tighter beam focus. For broader areal coverage and surface wetting, a higher liquid pressure (*P*_l_ = 0.4–0.5 MPa) combined with moderate gas pressure (*P*_g_ = 0.30–0.45 MPa) is more suitable.

## 5 Application of the Ultrasonic Atomization Dust Suppression Test

### 5.1 Design of the Ultrasonic Atomization Dust Suppression System

To verify the dust suppression effectiveness of ultrasonic atomization in the conveyor corridor of the Zhundong open-pit coal mine preparation plant in Xinjiang, an engineering-integrated ultrasonic atomization system was constructed, as shown in Figure 19. The system comprises three synergistic subsystems: gas supply, water supply, and dust measurement. The water circuit adopts a closed loop of a “water tank–pump–shut-off valve–pressure stabilizer–liquid flowmeter–spray chamber” to provide a stable liquid phase. The gas circuit consists of an “air compressor–pressure regulator–shut-off valve–gas flowmeter–spray chamber” to deliver the atomization drive. The measurement chain is equipped with real-time mass concentration analyzers (TSI 8533) and particle sizers (TSI 9306), along with total/respirable dust samplers (CCZ20­(A)), all calibrated and subjected to zero-point and drift checks, according to instrument specifications. Considering the conveyor belt width of 2 m, the spray chamber was designed with two swirling ultrasonic nozzles whose atomization angles and beam overlap ensured full coverage without blind zones.

**Figure 19:**
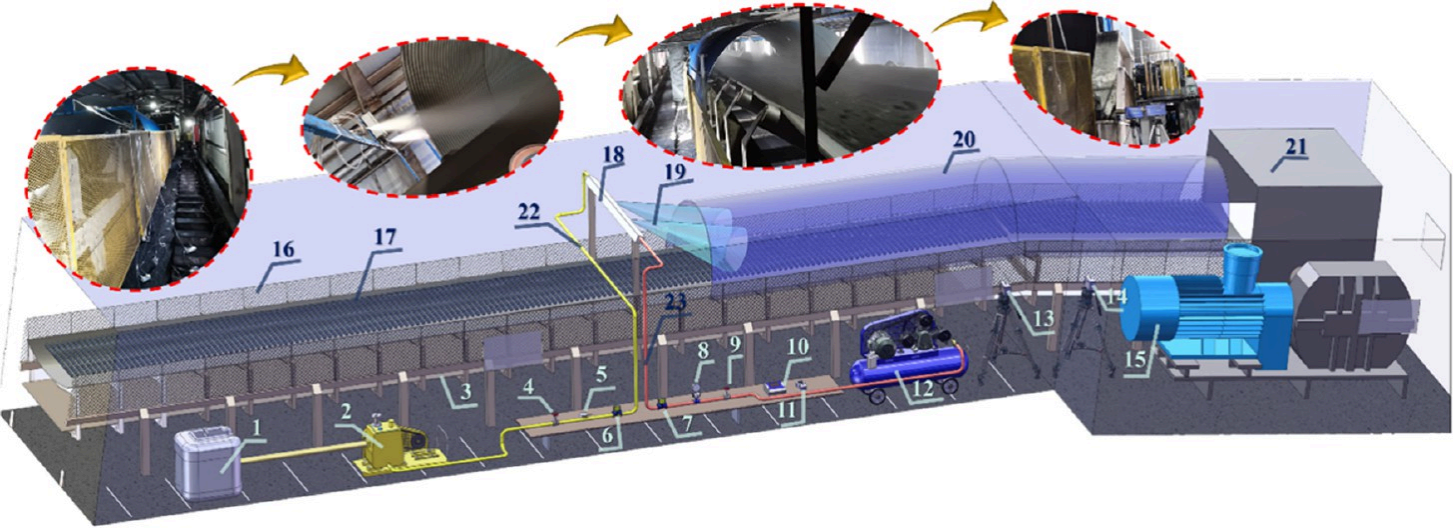
Field schematic of ultrasonic atomization dust removal in belt gallery of coal preparation plant. 1–Water tank; 2–water pump; 3–conveyor belt; 4–water-line shut-off valve; 5–water-line pressure stabilizer; 6–liquid flowmeter; 7–gas flowmeter; 8–pressure regulator; 9–gas-line shut-off valve; 10–dust monitor (model TSI 8533); 11–particle sizer (model TSI 9306); 12–air compressor; 13–total dust sampler (model CCZ20­(A)); 14–respirable dust sampler; 15–conveyor drive motor; 16–conveyor guard mesh; 17–raw coal; 18–ultrasonic atomization chamber; 19–atomization angle; 20–dust shield; 21–transfer-point protective cover; 22–liquid pipeline; 23–gas pipeline.

Combining experimental results, CFD-Post analysis, and field application, it was demonstrated that under operating conditions of *P*_g_ = 0.45 MPa and *P*_l_ = 0.1 MPa, a single nozzle produces dry-fog-scale droplets sufficient to achieve belt-surface coverage, effectively capturing dust while minimizing the risk of overwetting. The system is interlocked with the conveyor motor for synchronized start–stop control and incorporates PLC-based closed-loop regulation of pressure, flow feedback, and dust-threshold monitoring.

### 5.2 Comparative Analysis of the Dust Removal Effect of Ultrasonic Atomization

Figure 20 illustrates that following the application of swirling ultrasonic atomization in the conveyor corridor, both total dust and respirable dust concentrations decline markedly, exhibiting differentiated responses to gas and liquid pressure.

**Figure 20:**
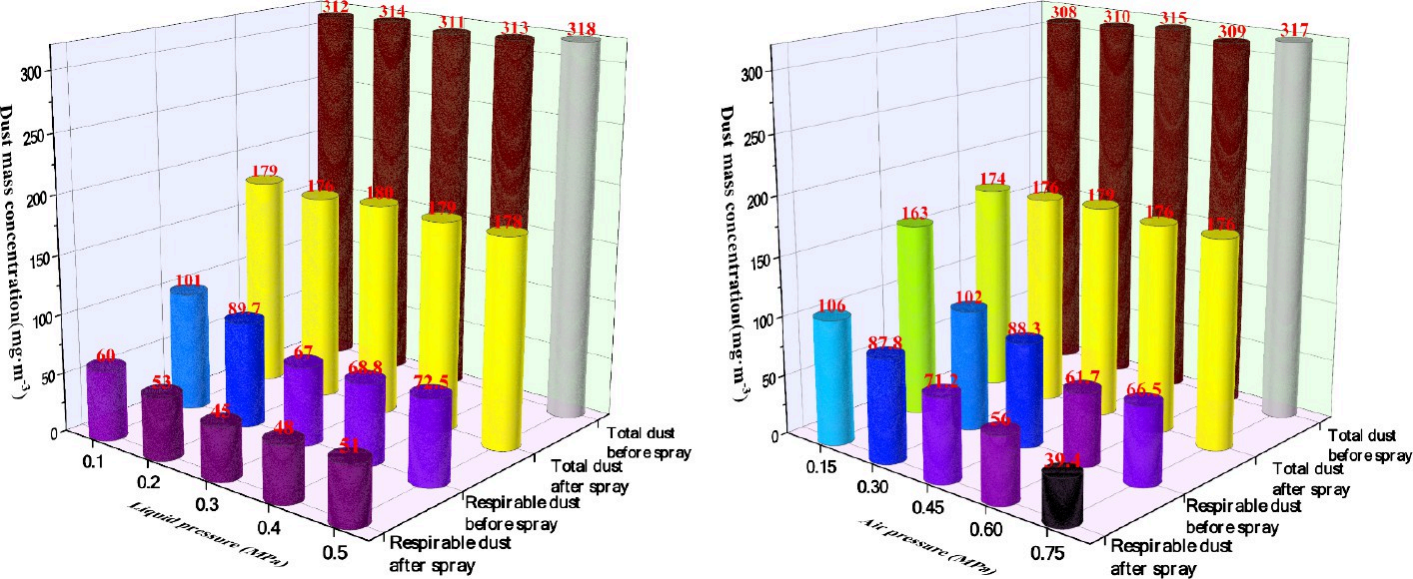
Effect of ultrasonic atomization with different gas–liquid pressures on dust suppression.

(1) With gas pressure *P*_g_ fixed at 0.45 MPa, dust suppression efficiency first increases and then decreases as liquid pressure *P*_l_ rises. The efficiency for total dust ranges from a minimum of 68% to a peak of 80%, while that for respirable dust varies from 61% to 75%. At the optimal liquid pressure of *P*_l_ = 0.3 MPa, total dust is reduced from 310 to 68 mg/m^3^, and respirable dust is reduced from 170 to 44 mg/m^3^. Beyond this point, however, increasing *P*_l_ enlarges droplet size, diminishes fine-particle capture, and reduces respirable dust efficiency.
(2) When liquid pressure is maintained at 0.1 MPa, both total and respirable dust suppression efficiencies improve steadily with rising gas pressure, with respirable dust showing a more pronounced upward trend. As *P*_g_ increases from 0.15 to 0.60 MPa, the total dust efficiency rises from 48% to 80%, while respirable dust efficiency improves from 39% to 79%. This confirms that higher gas pressure substantially enhances the dust suppression performance of the swirling ultrasonic nozzle, particularly against respirable dust.

In summary, gas pressure is the principal factor governing the effectiveness of ultrasonic atomization for dust control, while liquid pressure exhibits an optimal range between 0.2 and 0.3 MPa. To balance respirable dust capture with the need to avoid overwetting, it is recommended in practice to adopt *P*_g_ ≥ 0.6 MPa and *P*_l_ ∈ (0.2–0.3) MPa.

## 6 Conclusions

(1) The dust suppression efficiency of ultrasonic atomization is jointly governed by the gas and liquid flow rates. When the liquid flow is maintained at *Q*_l_ = 7.2 L/h, efficiency rises markedly with increasing gas flow, reaching nearly 90% capture efficiency for particles larger than 40 μm at *Q*_g_ = 300 L/min. Under constant gas flow of *Q*_g_ = 120 L/min, efficiency exhibits a “rise–fall” pattern with increasing liquid flow, with an optimal range of *Q*_l_ = 20–30 L/h. In this regime, coarse-particle capture remains high; however, further increases in liquid flow enlarge droplet size and accelerate premature settling, thereby reducing the efficiency of fine-particle capture.
(2) The gas-to-liquid momentum ratio is the decisive factor shaping ultrasonic atomization performance. As gas pressure increases from 0.15 to 0.75 MPa, gas flow rises from 25 to 136 L/min, the spray angle broadens from 14° to 57°, and the effective range extends from 122 to 281 cm. In contrast, although higher liquid pressure raises liquid flow, the resulting decrease in *Q*_g_/*Q*_l_ enlarges droplet size and diminishes secondary atomization efficiency, ultimately contracting the spray angle to less than 20° and shortening the range to below 150 cm. This highlights the dominant role of gas–liquid momentum competition in defining atomization characteristics.
(3) Gas and liquid pressures (*P*_g_ and *P*_l_) exert coupled effects on the flow ratio *Q*_l_/*Q*_g_ and droplet velocity *v*, expressed by the power-law relations *Q*_l_/*Q*_g_ = 6.16­(*P*_l_/*P*_g_)^−1.4^ and *v* = 0.176­(*P*_l_/*P*_g_)^−0.067^. For rapid near-field capture of respirable dust, a “high gas pressure–moderate liquid pressure” regime (*P*_g_ ≥ 0.6 MPa, *P*_l_ = 0.1–0.3 MPa) is recommended. For surface wetting and broad coverage, a higher liquid pressure (*P*_l_ = 0.4–0.5 MPa) in combination with moderate gas pressure (*P*_g_ = 0.30–0.45 MPa) proves more advantageous.
(4) Under optimal operating conditions, the swirling ultrasonic atomizer achieves dust suppression efficiencies of 80% for total dust and 75% for respirable dust. The dust-settling process can be delineated into three domains: droplet capture, coalescence–settling, and particle escape. This study elucidates the multiscale mechanisms of ultrasonic atomization for dust control, providing both theoretical foundations and practical guidance for the development of low-water-consumption, high-efficiency dust suppression technologies in mining environments.

The CFD model developed in this study does not resolve the internal gas–liquid coupling, swirling motion, or liquid-film breakup mechanisms inside the ultrasonic atomizing nozzle. Instead, an experimentally calibrated initial droplet spectrum is prescribed as the injection boundary condition. This modeling strategy is widely adopted in studies of ultrasonic and air-assisted atomization, as it effectively captures the transport and evolution of droplets in the external flow field, although it does not include the microscale atomization physics occurring within the resonant chamber.

## Data Availability

The data supporting this study are available within the manuscript.

## References

[1] Zazouli M. A., Dehbandi R., Mohammadyan M., Aarabi M., Dominguez A. O., Kelly F. J., Khodabakhshloo N., Rahman M. M., Naidu R. (2021). Physico-chemical
properties and reactive oxygen species generation by respirable coal
dust: Implication for human health risk assessment. Journal of Hazardous Materials.

[2] Zeng F. B., Jiang Z. A. (2023). Spatial and temporal
evolution of mine dust research:
visual knowledge mapping analysis in Web of Science from 2001 to 2021. Environ. Sci. Pollut. Res..

[3] Peng H., Peng Y., Nie W., Liu F., Xu C. (2025). Atomization
law and dust reduction effect of air-atomizing nozzles determined
by CFD and experiments. Energy.

[4] Wang Y. P., Jiang Z. G., Xu F., Wang J. Z., Zhang G. L., Zeng F. B. (2020). Study on parameters
of a new gas-water spray in ore
pass dedusting based on experiment and numerical simulation. Acs Omega.

[5] Wang J., Hong K., Song Y., Lu K. (2025). Multifactor influence
on smoke back-layering length of an upward single-slope tunnel with
a water spray system. International Journal
of Heat and Fluid Flow.

[6] Wang X., Wang P., Wu G., Li Y. (2025). Study of atomization
characteristics and dust suppression effect of micro-nano bubble enhanced
ultrasonic dry fog in excavation working face. Tunnelling and Underground Space Technology.

[7] Kim J. H., Lee H., Shin W. G. (2021). Horizontal
injection spray drying aerosol generator
using an ultrasonic nozzle with clean counter flow. J. Aerosol Sci..

[8] Peng H., Peng Y., Guo L., Liu F., Xu C. (2025). Effect of
structural parameters on atomization characteristics and dust reduction
performance of swirl nozzle. Powder Technol..

[9] Wang P., Zhang K., Liu R. (2019). Influence of air supply
pressure
on atomization characteristics and dust-suppression efficiency of
internal-mixing air-assisted atomizing nozzle. Powder Technol..

[10] Zhalehrajabi E., Rahmanian N., Zarrinpashne S., Balasubramanian P. (2014). Investigation
of the Growth of Particles Produced in a Laval Nozzle. Part. Sci. Technol..

[11] Yoon S. H., Kim D. Y., Kim D. K., Kim B. H. (2011). Effect
of nozzle geometry for swirl type twin-fluid water mist nozzle on
the spray characteristic. J. Mech. Sci. Technol..

[12] Li M., Tang J., Song X. Z., Qiu L. L., Yang H. Z., Li Z. (2022). Study on Multi-Factor
Optimization and Application for Water Mist
of a Wetting Dust Suppressant. ACS Omega.

[13] Saeedipour M., Schneiderbauer S., Plohl G., Brenn G., Pirker S. (2017). Multiscale
simulations and experiments on water jet atomization. International Journal of Multiphase Flow.

[14] Charinpanitkul T., Tanthapanichakoon W. (2011). Deterministic
model of open-space dust removal system
using water spray nozzle: Effects of polydispersity of water droplet
and dust particle. Sep. Purif. Technol..

[15] Simakov N. N. (2022). Calculation
of the interphase heat and mass transfer in a nozzle spray cone taking
into account the drag crisis and the heat- and mass-transfer crisis. Theor. Found. Chem. Eng..

[16] Han H., Wang P., Li Y., Liu R., Tian C. (2020). Effect of
water supply pressure on atomization characteristics and dust-reduction
efficiency of internal mixing air atomizing nozzle. Advanced Powder Technology.

[17] Xie Z., Zhao Z., Li D., Li F., Zhang C., Huang C., Xiao Y. (2023). Experimental study
on the atomization
characteristics and dust removal efficiency of a fan-shaped nozzle
for purifying working environment. Science of
The Total Environment.

[18] Tian Z., Xingyu C., Shaocheng G., Sheng L., Linquan T., Xinsheng M., Yuhao G. (2025). Experimental
study of the effect
of droplet motion velocity on the capture capacity of dust with different
characteristics. Chem. Eng. Res. Des..

[19] Nyabire Akanyange S., Nie W., Ilele Mwabaima F., Liu F., Niu W., Jiang S. Q., Zhang Y., Adom-Asamoah G., Luther Yeboah M., Qiu B. (2024). A systematic review
of the physiological and environmental impacts of coal dust and its
control technologies. Fuel.

[20] Beck T. W., Seaman C. E., Shahan M. R., Mischler S. E. (2018). Open-Air sprays
for capturing and controlling airborne float coal dust on longwall
faces. Mining Engineering.

[21] Wang Q., Wang D., Han F., Yang F., Sheng Y. (2020). Study and
application on foam-water mist integrated dust control technology
in fully mechanized excavation face. Process
Safety and Environmental Protection.

[22] Zhou G., Sun H., Wang Y., Liu B., Liu Y., Yao J., Liu Q., Sun B. (2025). Study on the
airflow-droplet synergistic dust control
and removal technology in fully mechanized excavation face: From the
perspective of coupling mechanism between atomized flow field and
turbulent air curtain. Process Safety and Environmental
Protection.

[23] Nie W., Yi S., Xu C., Zhang S., Peng H., Ma Q., Guo C., Cha X., Jiang C. (2023). Numerical simulation analysis of
a combined wind-fog dust removal device in return air roadways based
on an orthogonal test. Powder Technol..

[24] Wang Y., Jiang Z., Zhang F., Lu Y., Bao Y. (2022). Study on dust
diffusion characteristics of continuous dust sources and spray dust
control technology in fully mechanized working face. Powder Technol..

[25] Yang S., Ren J., Wan G., Ma F. (2025). Experimental
study on wind-fog coupling
dust suppression technology in tunnel drilling and blasting operations
based on CFD numerical simulation. Chem. Eng.
Res. Des..

[26] Mohan B. R., Jain R. K., Meikap B. C. (2008). Comprehensive analysis
for prediction of dust removal efficiency using twin-fluid atomization
in a spray scrubber. Sep. Purif. Technol..

[27] Jiang Z. A., Wang M., Chen J. S., Lin M. (2017). Atomization characteristics
and dust suppression mechanism of a gas-water nozzle. J. Harbin Inst. Technol..

[28] Ma S.-P., Kou Z.-M. (2005). Study on mechanism
of reducing dust by spray. J. China Coal Soc..

[29] Felis F., Tomas S., Vallet A., Amielh M., Anselmet F. (2020). Experimental
analysis of the flow characteristics of a pressure-atomised spray. International Journal of Heat and Fluid Flow.

[30] Xi X., Liu C., Pan Y., Zhang R., Song S., Xu S., Liu H. (2025). Numerical
investigation of in-nozzle cavitation and flow characteristics
in diesel engines using a multi-fluid quasi-VOF model coupled with
a cavitation model. International Journal of
Heat and Fluid Flow.

[31] Sun L. Y., Ge S. C., Liu S., Jing D. J., Chen X. (2022). Experimental and Molecular Dynamics
Simulation Study for Preferring
Coal Dust Wetting Agents. ACS Omega.

[32] Qi C. P., Weinell C. E., Dam-Johansen K., Wu H. (2023). Assessment of anticorrosion performance of zinc-rich epoxy coatings
added with zinc fibers for corrosion protection of steel. Acs Omega.

